# Cardiomyocyte proliferation and progenitor cell recruitment underlie therapeutic regeneration after myocardial infarction in the adult mouse heart

**DOI:** 10.1002/emmm.201201737

**Published:** 2013-01-29

**Authors:** Konstantinos Malliaras, Yiqiang Zhang, Jeffrey Seinfeld, Giselle Galang, Eleni Tseliou, Ke Cheng, Baiming Sun, Mohammad Aminzadeh, Eduardo Marbán

**Affiliations:** Cedars-Sinai Heart InstituteLos Angeles, CA, USA

**Keywords:** cardiac regeneration, cardiomyogenesis, cell therapy, fate mapping, myocardial infarction

## Abstract

Cardiosphere-derived cells (CDCs) have been shown to regenerate infarcted myocardium in patients after myocardial infarction (MI). However, whether the cells of the newly formed myocardium originate from the proliferation of adult cardiomyocytes or from the differentiation of endogenous stem cells remains unknown. Using genetic fate mapping to mark resident myocytes in combination with long-term BrdU pulsing, we investigated the origins of postnatal cardiomyogenesis in the normal, infarcted and cell-treated adult mammalian heart. In the normal mouse heart, cardiomyocyte turnover occurs predominantly through proliferation of resident cardiomyocytes at a rate of ∼1.3–4%/year. After MI, new cardiomyocytes arise from both progenitors as well as pre-existing cardiomyocytes. Transplantation of CDCs upregulates host cardiomyocyte cycling and recruitment of endogenous progenitors, while boosting heart function and increasing viable myocardium. The observed phenomena cannot be explained by cardiomyocyte polyploidization, bi/multinucleation, cell fusion or DNA repair. Thus, CDCs induce myocardial regeneration by differentially upregulating two mechanisms of endogenous cell proliferation.

→See accompanying article http://dx.doi.org/10.1002/emmm.201202345

## INTRODUCTION

The concept of the adult mammalian heart as a dynamic organ capable of limited endogenous regeneration has been convincingly established (Bergmann et al, 2009). However, estimated rates of turnover vary wildly (Kajstura et al, 2010) and the underlying cellular mechanisms remain unclear (Laflamme & Murry, 2011; Steinhauser & Lee, 2011). It is still unknown whether the adult mammalian heart generates new cardiomyocytes through myogenic differentiation of endogenous stem cells [as has been described in the normal (Hosoda et al, 2009), infarcted (Hsieh et al, 2007) and cell-treated (Hatzistergos et al, 2010; Loffredo et al, 2011) heart] or through proliferation of resident cardiomyocytes (Boström et al, 2010) [as observed in zebrafish (Kikuchi et al, 2010) and neonatal mice (Porrello et al, 2011)].

Cardiosphere-derived cells (CDCs) (Smith et al, 2007) have been shown to regenerate functional heart muscle in patients post-myocardial infarction (MI) in the recently reported CADUCEUS study (Makkar et al, 2012). Nevertheless, the cellular origin of the newly formed myocardium remains unknown. Using a genetic fate mapping approach (to specifically mark resident cardiomyocytes and their progeny) in combination with long-term BrdU pulsing and assays that minimize potential confounding factors, we investigated the cellular origins of postnatal cardiomyogenesis in the normal, infarcted and CDC-treated adult mammalian heart.

## RESULTS

### CDCs stimulate resident cardiomyocyte cycling after myocardial infarction

**Figure 1:**
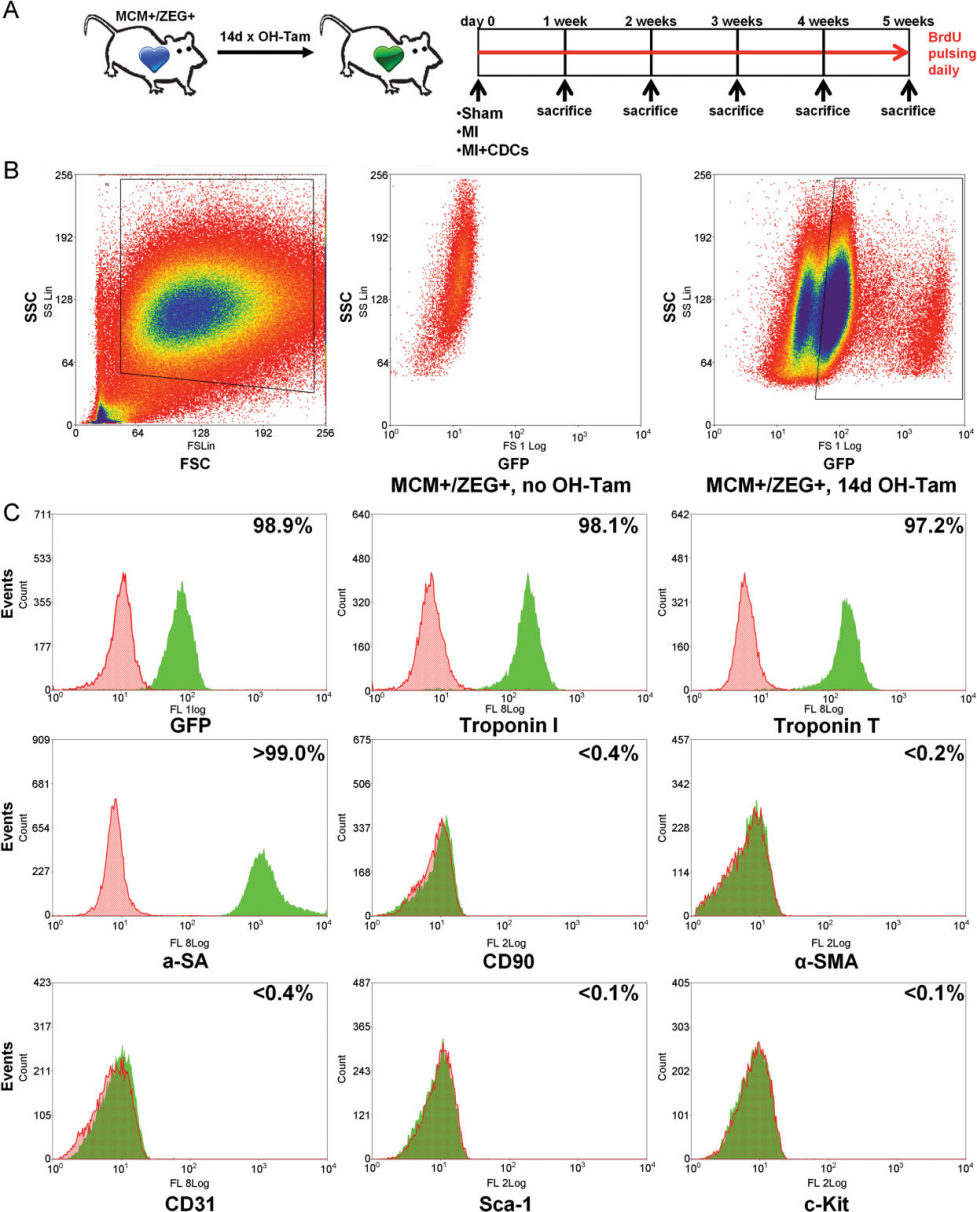
Isolation of resident cardiomyocytes Study schematic. A tamoxifen-inducible cardiomyocyte-specific genetic fate mapping approach was employed, utilizing the MerCreMEr/ZEG mouse strain.Fluorescence-activated cell sorting of enzymatically dispersed myocardial cell preparations obtained from 4-OH Tamoxifen pulsed and non-pulsed bitransgenic (control) mice. Cells were initially gated on the basis of size (Forward scatter; FSC) and granularity (Side scatter; SSC) and subsequently sorted based on expression of GFP, a specific marker for endogenous cardiomyocytes. Boxes denote the boundaries of sorted populations.Flow cytometric analysis for assessment of purity of FACS-sorted GFP^+^ cardiomyocytes. Markers include troponin T, troponin I, and α-sarcomeric actinin (for cardiomyocytes), CD90 (for mesenchymal cells and fibroblasts), α-smooth muscle actin (α-SMA) (for smooth muscle cells), CD31 (for endothelial cells), c-Kit and Sca-1 (for progenitor cells).

We used an inducible fate mapping approach which provides efficient and highly specific cardiomyocyte labelling (Hsieh et al, 2007; Loffredo et al, 2011; Zhang et al, 2010). Bitransgenic mice were generated by crossbreeding female transgenic B6129-Tg(Myh6-cre/Esr1)1 Jmk/J (hereafter referred to as MerCreMer) mice with male B6.Cg-Tg(ACTB-Bgeo/GFP)21Lbe/J (hereafter referred to as ZEG) reporter mice. MerCreMer mice carry a fusion transgene of Cre recombinase flanked by Mer (mutated estrogen receptor ligand binding domains), driven by the cardiac α-myosin heavy chain promoter (encoded by Myh6); thus the Cre recombinase activity is tamoxifen-sensitive and cardiomyocyte-specific. ZEG reporter mice carry a lacZ transgene flanked by LoxP sites, followed by stop codons and then the GFP gene; therefore, upon excision of the LoxP sites and stop codons mediated by Cre recombinase activity, the reporter switches to GFP (driven by the β-actin promoter), permanently and specifically marking resident cardiomyocytes and their progeny as GFP^+^. No spontaneous expression of GFP was detected in non-tamoxifen-pulsed bitransgenic animals [confirming previous studies reporting very low rates of leakage in MerCreMer/ZEG mice (Hsieh et al, 2007)], while pulsed mice exhibited robust GFP expression (Fig 1B, Supporting Information Fig 1).

After 14 days of daily 4-OH-Tamoxifen administration [which resulted in a 79.2% (±7.1%) labelling efficiency of cardiomyocytes (Supporting Information Fig 1C), confirming previous reports (Hsieh et al, 2007; Loffredo et al, 2011)], 6–8 week old bitransgenic MerCreMer/ZEG mice were randomly assigned to three groups: (a) sham surgery; (b) induction of MI by permanent ligation of the left anterior coronary artery (LAD); (c) induction of MI followed by intramyocardial injection of mouse CDCs (2 × 10^5^, grown from wild-type animals) into the infarct border zone. Mice were pulsed daily with BrdU and sacrificed at weekly intervals up to 5 weeks (Fig 1A). Hearts were explanted and enzymatically dissociated by retrograde collagenase perfusion. Fluorescence-activated cell sorting (FACS) of the isolated cells for large size (Diez & Simm, 1998) and robust GFP expression (Fig 1B) yielded highly pure cardiomyocytes (cells positive for GFP = 98.9%, for Troponin *T* = 97.2%, for Troponin *I* = 98.1% and for α-sarcomeric actinin [αSA] > 99%) with minimal contamination by mesenchymals cells or fibrobalsts (CD90^+^ cells: <0.4%), smooth muscle cells (α-smooth muscle actin^+^ cells: <0.2%) or endothelial cells (CD31^+^ cells: <0.4%; Fig 1C). Neither c-Kit^+^ nor Sca-1^+^ cells were detectable in the GFP^+^ sorted cell population (Fig 1C), verifying that the sorted cells are mature cardiomyocytes, not partially differentiated cardiac progenitor cells (Hsieh et al, 2007; Zhang et al, 2010).

**Figure 2:**
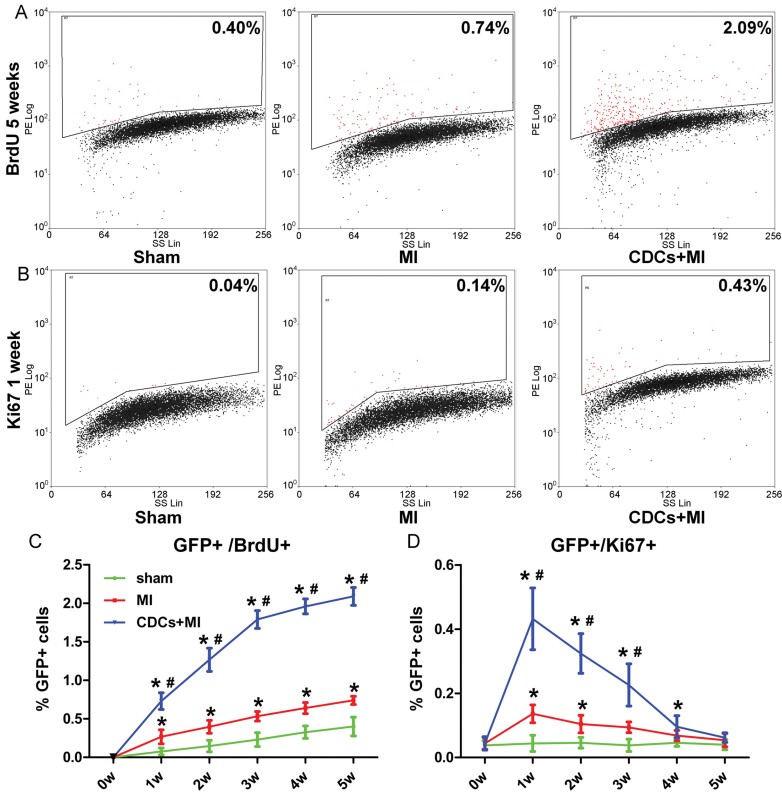
Resident cardiomyocyte turnover in the adult mouse heart assessed by flow cytometry **A–D.** Flow cytometric analysis of GFP^+^ sorted cardiomyocytes for BrdU incorporation (**A**,**C**) and Ki67 expression (**B**,**D**) reveals that the young adult heart contains a small pool of cycling resident cardiomyocytes that increases after MI and is upregulated by CDC therapy. Numbers in flow cytometry plots indicate averages for groups. Red dots indicate BrdU/Ki67^+^ resident cardiomyocytes while black dots indicate BrdU/Ki67^−^ resident cardiomyocytes (colour gating has been applied to the images) (*p < 0.05 compared to sham; ^#^*p* < 0.05 compared to MI, *n* = 3–5/group/timepoint). All error bars represent SDs. One-way ANOVA followed by LSD *post hoc* test was used for statistical analysis (C: MI *vs* sham: 1w *p* = 0.035, 2w *p* = 0.031, 3w *p* = 0.007, 4w *p* = 0.004, 5w *p* = 0.006; CDCs *vs* sham: 1w *p* < 0.001, 2w *p* < 0.001, 3w *p* < 0.001, 4w *p* < 0.001, 5w *p* < 0.001; CDCs *vs* MI: 1w *p* = 0.001, 2w *p* < 0.001, 3w *p* < 0.001, 4w *p* < 0.001, 5w *p* < 0.001; D: MI vs sham: 1w *p* = 0.031, 2w *p* = 0.041, 3w *p* = 0.049; CDCs *vs* sham: 1w *p* < 0.001, 2w *p* < 0.001, 3w *p* < 0.001, 4w *p* = 0.005; CDCs *vs* MI: 1w *p* < 0.001, 2w *p* < 0.001, 3w *p* < 0.001; all other *p* = ns).

**Figure 3:**
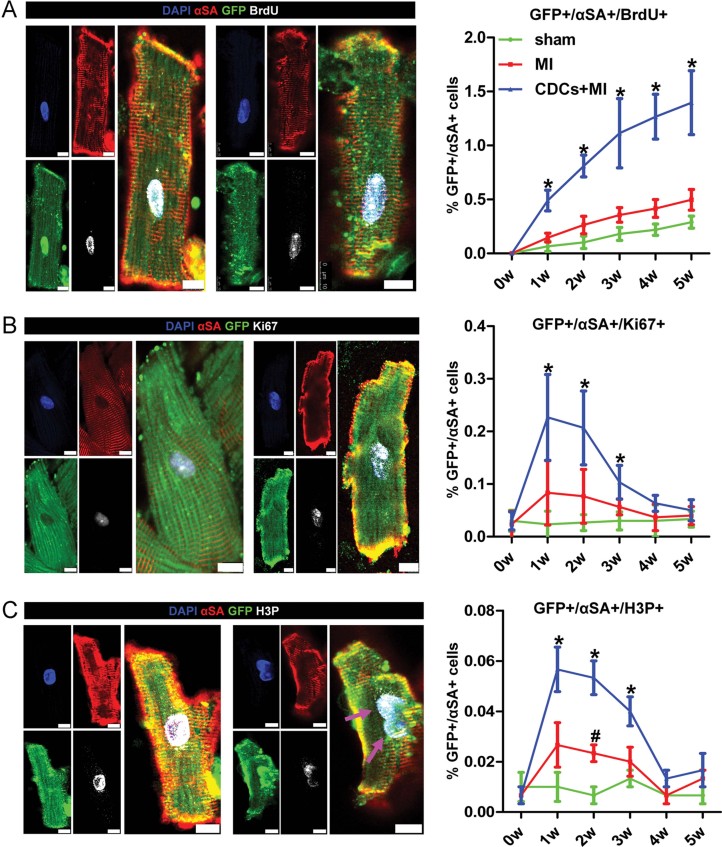
Resident cardiomyocyte turnover in the adult mouse heart assessed by immunocytochemistry **A–C.** Immunocytochemistry of enzymatically dissociated cardiomyocytes for GFP, αSA and BrdU (**A**), Ki67 (**B**), H3P (**C**) reveals that the young adult heart contains a small pool of cycling resident cardiomyocytes that increases after MI and is upregulated by CDC therapy. Arrows in (**C**) show an example of karyokinesis (*p < 0.05 compared to sham, MI, ^#^*p* < 0.05 compared to sham only, *n* = 3/group/timepoint). All error bars represent SDs. One-way ANOVA followed by LSD *post hoc* test was used for statistical analysis (A: CDCs *vs* sham: 1w *p* < 0.001, 2w *p* < 0.001, 3w *p* = 0.001, 4w *p* < 0.001, 5w *p* < 0.001; CDCs *vs* MI: 1w *p* = 0.001, 2w *p* < 0.001, 3w *p* = 0.003, 4w *p* < 0.001, 5w *p* = 0.001; B: CDCs *vs* sham: 1w *p* = 0.006, 2w *p* = 0.005, 3w *p* = 0.008; CDCs *vs* MI: 1w *p* = 0.027, 2w *p* = 0.021, 3w *p* = 0.046; C: MI *vs* sham 2w *p* = 0.020; CDCs *vs* sham: 1w *p* = 0.009, 2w *p* < 0.001, 3w *p* = 0.011; CDCs *vs* MI: 1w *p* = 0.038, 2w *p* = 0.003, 3w *p* = 0.046; all other *p* = ns). All scale bars: 10 µm.

Flow cytometry of BrdU and Ki67 in FACS-sorted GFP^+^ cardiomyocytes revealed that the normal heart contains a small fraction of cycling endogenous cardiomyocytes (BrdU^+^: 0.08 ± 0.05% after the 1st week of BrdU pulsing, 0.4 ± 0.12% after 5 weeks of BrdU pulsing; Ki67^+^: 0.04 ± 0.03%). The low but measurable rate of basal cycling is consistent with some reports of cardiomyocyte turnover in the young adult heart (Bergmann et al, 2009; Soonpaa & Field, 1997), but not others (Kajstura et al 2010; Walsh et al, 2010). Tissue injury results in increased cardiomyocyte cycling, primarily during the first 3 weeks post-MI (BrdU^+^: 0.27 ± 0.09% after the 1st week of BrdU pulsing, 0.74 ± 0.05% after 5 weeks of BrdU pulsing; Ki67^+^: 0.14 ± 0.03%). Both the low rate of cardiomyocyte cycling under basal conditions, as well as the increase after injury, are notable. However, the most surprising finding is the amplification of cardiomyocyte cycling by cell therapy: the number of BrdU-incorporating preformed cardiomyocytes increases approximately threefold relative to MI (and approximately ninefold over basal levels) to 0.73 ± 0.11% after the 1st week of BrdU pulsing (2.09 ± 0.12% after 5 weeks of BrdU pulsing). Likewise, the Ki67^+^ percentage rises to 0.43 ± 0.09% 1 week after CDC administration (Fig 2A–D, Supporting Information Fig 2). The differences were greatest in the first 3 weeks post-injury. Immunocytochemistry of enzymatically dissociated cardiomyocytes (GFP^+^, αSA^+^) for BrdU, Ki67 and H3P (a marker of karyokinesis) confirmed these results (Fig 3).

It has been reported that BrdU can be toxic to tissues with high proliferation rates, such as the skin and the gastrointestinal tract (Kimbrough et al, 2011) and that exposure to BrdU can influence the proliferation of murine hepatic and renal cells (Weghorst et al, 1991). However, no toxic effects were observed in long-term rodent studies of BrdU (Jecker et al, 1997). To exclude a potential effect of long-term BrdU administration on the cycling rates of cardiomyocytes, 4-OH-Tamoxifen pulsed bitransgenic mice were randomized to undergo sham surgery, MI or MI followed by CDC injection, without receiving BrdU. One and five weeks later, hearts were enzymatically dissociated by retrograde collagenase perfusion and isolated cardiomyocytes underwent immunocytochemistry for GFP, αSA and Ki67. No significant differences in the percentage of Ki67^+^/GFP^+^ cardiomyocytes were detected between mice that received BrdU (Fig 3B) and mice that did not receive BrdU (Supporting Information Fig 3) at 1 and 5 weeks, ruling out a major effect of long-term BrdU administration on the cycling rates of resident cardiomyocytes.

### Cycling resident cardiomyocytes are smaller, more often mononucleated and reside primarily in the peri-infarct area

**Figure 4:**
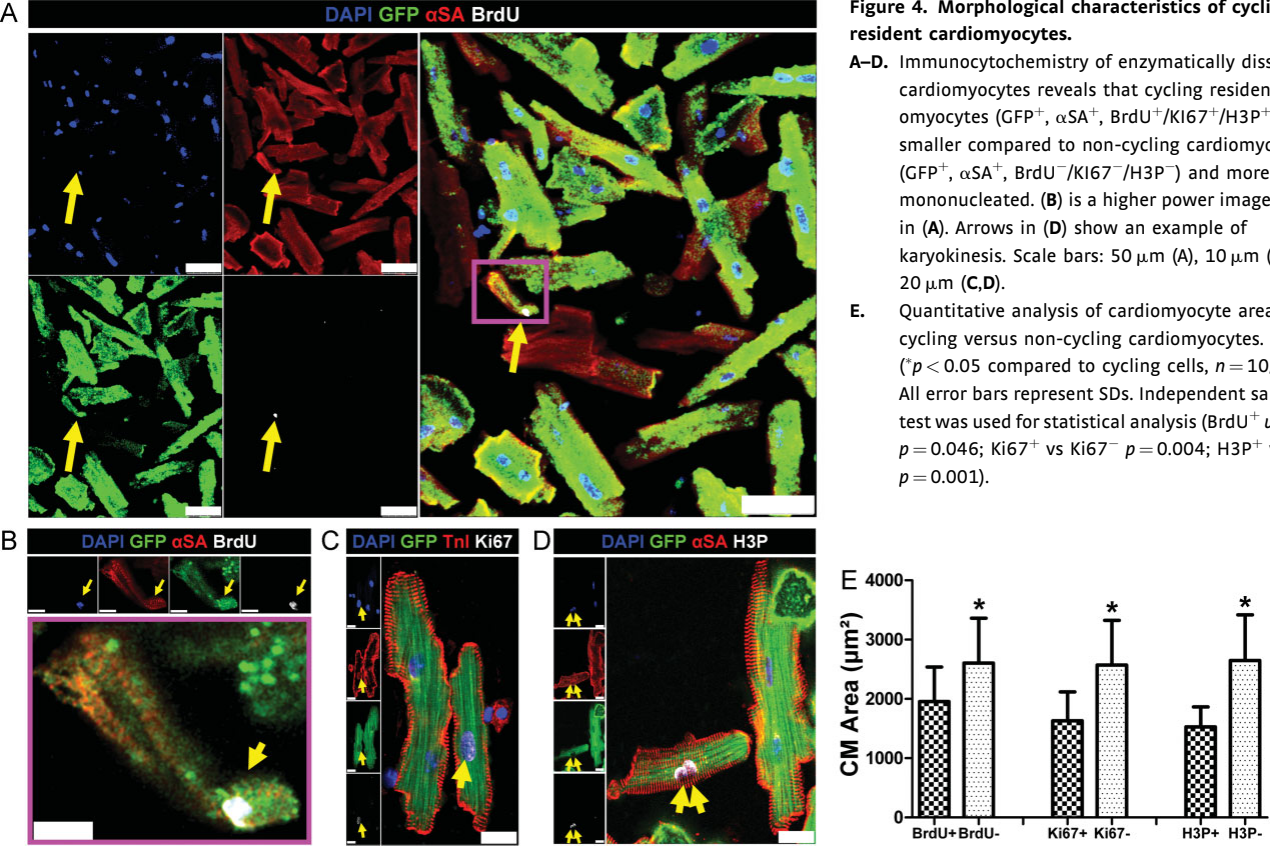
Morphological characteristics of cycling resident cardiomyocytes **A–D.** Immunocytochemistry of enzymatically dissociated cardiomyocytes reveals that cycling resident cardiomyocytes (GFP^+^, αSA^+^, BrdU^+^/KI67^+^/H3P^+^) were smaller compared to non-cycling cardiomyocytes (GFP^+^, αSA^+^, BrdU^−^/KI67^−^/H3P^−^) and more often mononucleated. (**B**) is a higher power image of inset in (**A**). Arrows in (**D**) show an example of karyokinesis. Scale bars: 50 µm (A), 10 µm (**B**), 20 µm (**C**,**D**).**E.** Quantitative analysis of cardiomyocyte area in cycling versus non-cycling cardiomyocytes. (*p < 0.05 compared to cycling cells, *n* = 10/group). All error bars represent SDs. Independent samples *t*-test was used for statistical analysis (BrdU^+^ *vs* BrdU^−^ *p* = 0.046; Ki67^+^ vs Ki67^−^ *p* = 0.004; H3P^+^ vs H3P^−^ *p* = 0.001).

**Figure 5:**
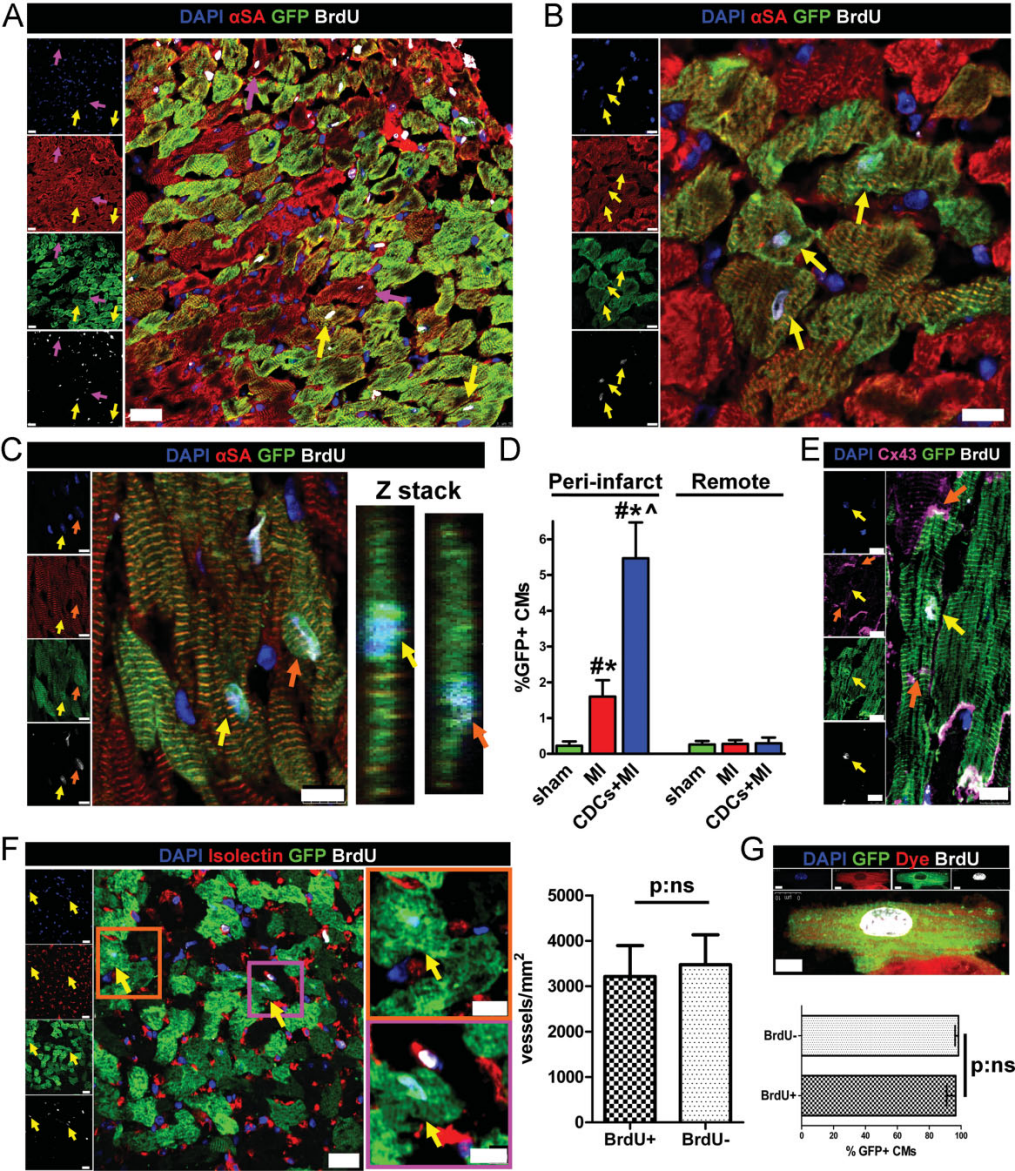
Cycling resident cardiomyocytes in the peri-infarct area **A–C.** BrdU^+^/GFP^+^ resident cardiomyocytes in the peri-infarct area are indicated by yellow arrows in (**A**–**C**) and orange arrows in (**C**). Purple arrows in (**A**) show BrdU^+^/GFP^−^ cardiomyocytes. Z-stacks in (**C**) reveal that the BrdU^+^ nuclei belong to cardiomyocytes.**D.** Quantification of the rates of cycling endogenous cardiomyocytes by immunohistochemistry reveals that ∼90% of GFP^+^/αSA^+^/BrdU^+^ cardiomyocytes were located in the peri-infarct area after MI and CDC therapy (*p < 0.05 compared to remote, ^#^*p* < 0.05 compared to sham, ∧*p* < 0.05 compared to MI, *n* = 3–5/group). One-way ANOVA followed by LSD *post hoc* test and independent samples *t*-test were used for statistical analysis (MI *vs* sham *p* = 0.039; CDCs *vs* sham *p* < 0.001; CDCs *vs* MI *p* < 0.001; MI peri-infarct *vs* remote *p* = 0.008; CDCs peri-infarct *vs* remote *p* = 0.001; all other *p* = ns).**E.** Immunohistochemistry reveals that BrdU^+^ resident cardiomyocytes (yellow arrows) are connected with gap junctions (orange arrows) to neighbouring BrdU^−^ cardiomyocytes.**F.** Vessel density was similar in areas of the border zone that contained BrdU^+^/GFP^+^ resident cardiomyocytes (yellow arrows; images on the right are high power images of insets on left) compared to areas that did not contain BrdU^+^ cardiomyocytes (*n* = 3/group). Independent samples *t*-test was used for statistical analysis.**G.** After *ex vivo* retrograde perfusion with Celltracker RED dye the percentage of Celltracker RED^+^ cardiomyocytes did not differ between BrdU^+^ and BrdU^−^ GFP^+^ cardiomyocytes, verifying that cycling myocytes are normally perfused (*n* = 3/group). Independent samples *t*-test was used for statistical analysis. All error bars represent SDs. Scale bars: 20 µm (**A**,**F**), 10 µm (**B**,**C**,**E**,**F[**high power],**G**).

Immunocytochemistry of isolated cells revealed that cycling (BrdU^+^ or Ki67^+^ or H3P^+^) GFP^+^/αSA^+^ cardiomyocytes were smaller (Fig 4A–E) and more often mononucleated (Figs 4A–C and 9A–C), compared to non-cycling (BrdU^−^, or Ki67^−^ or H3P^−^) GFP^+^/αSA^+^ cardiomyocytes, consistent with previous findings (Chen et al, 2007). Flow cytometric analysis confirmed that cycling endogenous cardiomyocytes (BrdU^+^/GFP^+^ cells) were smaller (decreased time of flight, decreased forward scatter area) and less granular/complex (decreased side scatter area) compared to non-cycling endogenous cardiomyocytes (BrdU^−^/GFP^+^; Supporting Information Fig 4). Tissue immunohistochemistry revealed that ∼90% of cycling resident cardiomyocytes after MI and CDC therapy were located in the peri-infarct area [Fig 5A–D and F; defined as the area within one low-power field from the edges of the scar, but not including the scar itself (Supporting Information Fig 5)]. Furthermore, BrdU^+^ cardiomyocytes appear to be structurally integrated with the surrounding myocardium, as they are connected by gap junctions to neighbouring non-cycling myocytes (Fig 5E). Within the border zone, vessel densities were comparable in areas adjacent to BrdU^+^ cardiomyocytes as compared to those remote from BrdU^+^ cardiomyocytes (Fig 5F). To unequivocally show that cycling myocytes are normally perfused, we performed *ex vivo* retrograde perfusion of hearts (obtained from BrdU-pulsed bitransgenic mice) with a fluorescent dye (Celltracker RED), followed by enzymatic dissociation. The vast majority (29 of 30) BrdU^+^ cardiomyocytes were positive for Celltracker RED; the percentage of Celltracker RED^+^ cardiomyocytes did not differ between BrdU^+^ and BrdU^−^ GFP^+^ cardiomyocytes (Fig 5G). Given that access to infused Celltracker RED is haematogenous, we conclude that cycling myocytes are normally perfused.

### CDCs upregulate expression of genes associated with cell-cycle progression in resident cardiomyocytes

**Figure 6:**
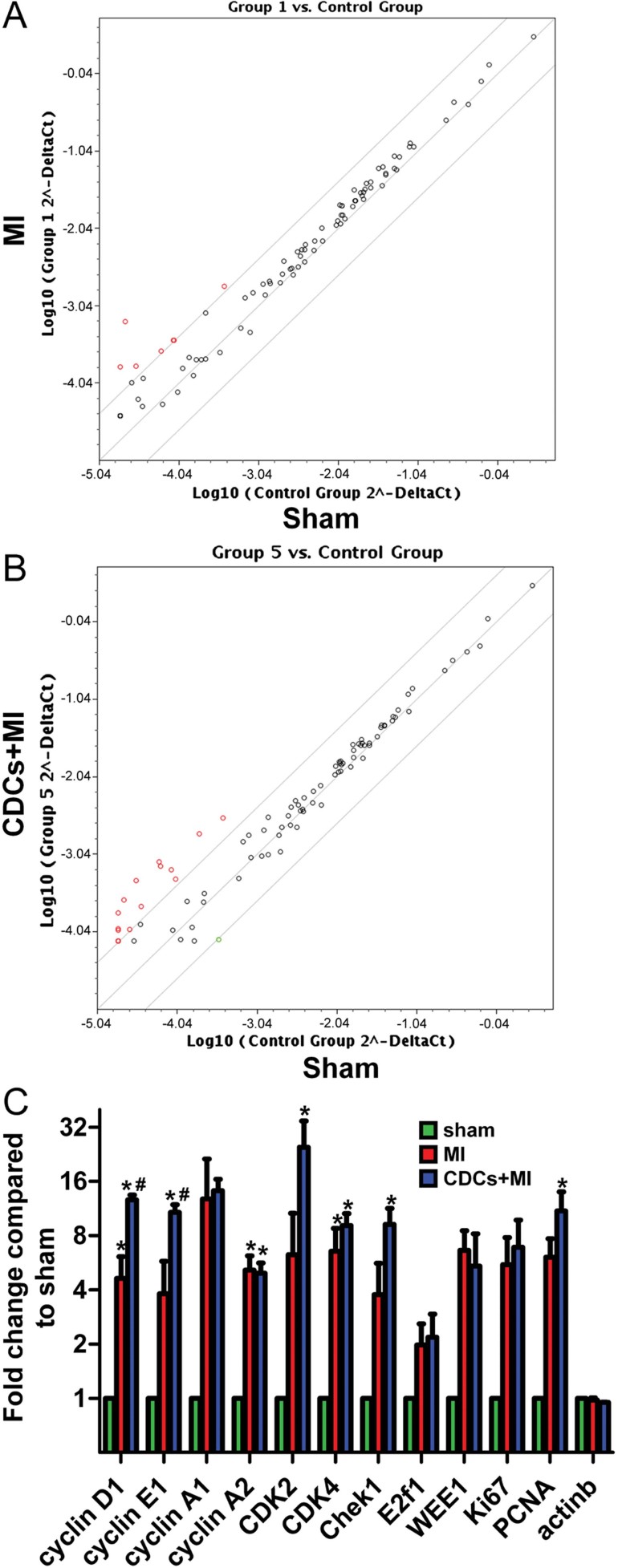
Upregulation of cell-cycle associated genes in resident cardiomyocytes **A, B.** Representative scatter plots of PCR microarray analysis of RNA isolated from GFP^+^ FACS-sorted cardiomyocytes for cell-cycle associated genes showing increased expression of positive cell-cycle regulators post-MI (red dots) compared to sham-operated animals (**A**), changes which are further upregulated after CDC therapy (**B**).**C.** Quantitative analysis of PCR microarray data. Results are presented as fold change compared to sham and were calculated using the ΔΔ*C*_t_ method. B-Actin was used as a control. (*p < 0.05 compared to sham; ^#^*p* < 0.05 compared to MI, *n* = 3/group). All error bars represent SDs. One-way ANOVA followed by LSD *post hoc* test was used for statistical analysis (cyclin D1: MI *vs* sham *p* = 0.047, CDCs *vs* sham *p* = 0.001, CDCs *vs* MI *p* = 0.004; cyclin E1: CDCs *vs* sham *p* = 0.005, CDCs *vs* MI *p* = 0.02; cyclin A2: MI *vs* sham *p* = 0.009, CDCs *vs* sham *p* = 0.016, CDK4: MI *vs* sham *p* = 0.047, CDCs *vs* sham *p* = 0.019, Chek1: CDCs *vs* sham *p* = 0.014, PCNA: CDCs *vs* sham *p* = 0.009; all other *p* = ns).

In order to detect changes in gene expression, we isolated RNA from FACS-sorted GFP^+^ cardiomyocytes and performed PCR microarray analysis for cell-cycle associated genes. We found that MI upregulated several genes associated with cell-cycle progression in resident cardiomyocytes, the expression of which was further amplified by therapy with CDCs (Fig 6). These genes include ones that orchestrate the G0/G1 transition (Cyclin D1, Cyclin-Dependent Kinase 4), the G1/S transition (Cyclin E, Cyclin-Dependent Kinase 2) and the G2/M transition (Cyclin A1-2, E2F1) (Li & Brooks, 1999; Pasumarthi & Field, 2002). Most of these genes have been shown to be upregulated in the embryonic and neonatal heart (Walsh et al, 2010), which are known to be capable of cardiomyocyte hyperplasia and robust regeneration post-injury (Porrello et al, 2011).

### Origins of regenerative cardiomyogenesis

**Figure 7:**
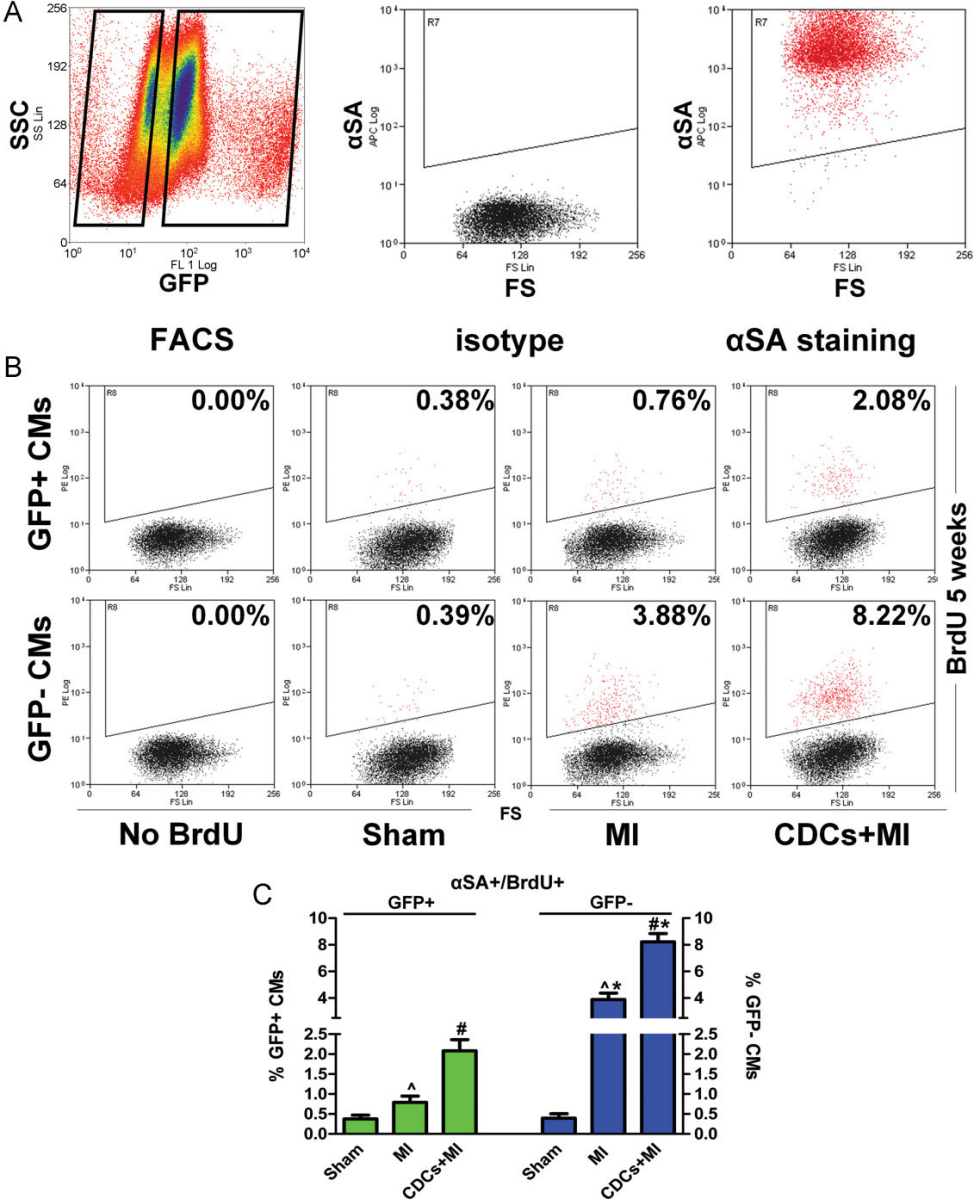
Origins of postnatal cardiomyogenesis in the adult mouse heart by flow cytometry **A.** FACS-sorted GFP^+^ and GFP^−^ cardiomyocytes were subsequently stained for αSA. Only αSA^+^ cells were examined.**B, C.** Flow cytometric analysis of GFP^+^/αSA^+^ and GFP^−^/αSA^+^ cardiomyocytes for BrdU incorporation reveals that, in the normal mouse heart, cardiomyocyte turnover occurs exclusively through proliferation of adult cardiomyocytes. After MI, cardiomyocyte proliferation is upregulated, while progenitor cells also contribute to the replacement of lost cardiomyocytes. CDCs amplify both stem cell-mediated myocyte replenishment and adult cardiomyocyte proliferation. Numbers in flow cytometry plots indicate averages for groups. Red dots indicate BrdU^+^ while black dots indicate BrdU^−^ cardiomyocytes (colour gating has been applied to the images). (*p < 0.05 compared to GFP^+^ cardiomyocytes; ^#^*p* < 0.05 compared to MI and sham; ∧*p* < 0.05 to sham; *n* = 5/group). All error bars represent SDs. One-way ANOVA followed by LSD *post hoc* test and independent samples *t*-test were used for statistical analysis (GFP^+^: MI *vs* sham: *p* = 0.01, CDCs *vs* sham *p* < 0.001, CDCs *vs* MI *p* < 0.001; GFP^−^: MI *vs* sham: *p* < 0.001, CDCs *vs* sham *p* < 0.001, CDCs *vs* MI *p* < 0.001; GFP^+^ *vs* GFP^−^ MI *p* < 0.001, GFP^+^ *vs* GFP^−^ CDCs *p* < 0.001; all other *p* = ns).

We next sought to determine the relative contributions of adult cardiomyocyte proliferation and cardiomyogenic differentiation of endogenous stem cells in postnatal cardiomyogenesis. 4-OH-Tamoxifen-pulsed 6–8 week-old bitransgenic mice were randomized to undergo: (a) sham surgery; (b) induction of MI by permanent LAD ligation; or (c) induction of MI followed by intramyocardial injection of mouse CDCs (2 × 10^5^, grown from wild-type animals) into the infarct border zone. Mice were subsequently pulsed with daily BrdU injections for 5 weeks, at which point hearts were explanted and enzymatically dissociated. We used FACS-sorting (gating on large cells) to isolate GFP^+^ and GFP^−^ cardiomyocytes (Fig 7A), which subsequently underwent flow cytometry for αSA expression and BrdU incorporation. Only αSA^+^ cells were examined (Fig 7A), in order to assure the purity of the GFP^−^ fraction, and to avoid confounding results arising from contamination by GFP^−^ non-myocytes. By comparing the rates of BrdU incorporation in GFP^+^ versus GFP^−^ cardiomyocytes, we were able to calculate the absolute rates and relative magnitudes of induced secondary cardiomyocyte proliferation and cardiomyogenic differentiation of recruited endogenous stem cells in postnatal cardiomyogenesis in the normal, infarcted and CDC-treated hearts. Taking into account that GFP only marks preformed resident cardiomyocytes, equal rates of BrdU incorporation in GFP^+^ and GFP^−^ myocytes would translate to no significant contribution of endogenous progenitors to the myocyte pool during the course of BrdU pulsing (as the cardiomyocytes arising from progenitors would by default be GFP^−^). Increased rates of BrdU incorporation in the GFP^−^ myocyte fraction would translate to myogenic differentiation of progenitors during the course of BrdU pulsing.

We found that, in the normal mouse heart, rates of BrdU incorporation (after 5 weeks of daily administration) were similar in the GFP^+^/αSA^+^ (0.38 ± 0.09%) and GFP^−^/αSA^+^ fractions (0.39 ± 0.11%, *p* = 0.79; Fig 7B and C). Thus, in the normal adult mouse heart, cardiomyocyte turnover occurs mainly through proliferation of resident myocytes, without any significant contribution by endogenous progenitors. After MI, the rate of BrdU incorporation increased in both GFP^+^/αSA^+^ and GFP^−^/αSA^+^ fractions compared to that observed in the normal heart (confirming the increase in cardiomyocyte cycling post-MI); however, the percentage of BrdU^+^/GFP^−^/αSA^+^ myocytes (3.88 ± 0.49%) was higher compared to the percentage of BrdU^+^/GFP^+^/αSA^+^ myocytes (0.76 ± 0.18%, *p* < 0.001; Fig 7B and C). Therefore, in the post-MI setting, progenitor cells also contribute to the replacement of lost cardiomyocytes; both the lack of a progenitor cell contribution at baseline, and its appearance post-MI, confirm previous reports in the same model (Hsieh et al, 2007; Loffredo et al, 2011). Therapy with CDCs increased BrdU incorporation in both GFP^+^/αSA^+^ and GFP^−^/αSA^+^ fractions compared to MI; the increase was disproportionally greater in GFP^−^/αSA^+^ cardiomyocytes (8.22 ± 0.63% *vs* 2.08 ± 0.27%, *p* < 0.001; Fig 7B and C). Thus, CDCs amplify both stem cell-mediated myocyte replenishment and adult cardiomyocyte proliferation. It should be noted that the measured rate of BrdU incorporation in the GFP^+^/αSA^+^ cardiomyocytes (Fig 7B and C) was virtually identical to that observed when FACS-sorted GFP^+^ cells (without staining and gating on αSA^+^ cardiomyocytes) were analysed (Fig 2A and C), confirming that the analysed FACS-sorted GFP^+^/BrdU^+^ cells were indeed cardiomyocytes, and not contaminating BrdU^+^ non-myocytes.

**Figure 8:**
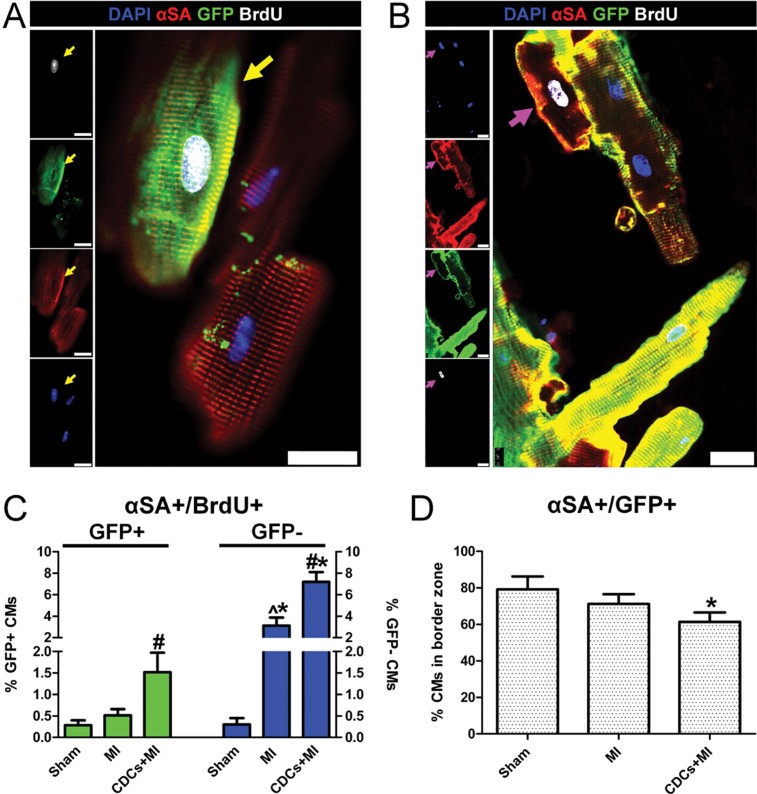
Origins of postnatal cardiomyogenesis in the adult mouse heart by immunocytochemistry **A–C.** Immunocytochemistry of enzymatically dissociated cardiomyocytes for GFP, αSA and BrdU reveals that, in the normal mouse heart, cardiomyocyte turnover occurs exclusively through proliferation of adult cardiomyocytes. After MI, cardiomyocyte proliferation is upregulated, while progenitor cells also contribute to the replacement of lost cardiomyocytes. CDCs amplify both stem cell-mediated myocyte replenishment and adult cardiomyocyte proliferation. Arrows in (**A**) indicate a GFP^+^/BrdU^+^ cardiomyocyte. Arrows in (**B**) show a GFP^−^/BrdU^+^ cardiomyocyte. (*p < 0.05 compared to GFP^+^ cardiomyocytes; ^#^*p* < 0.05 compared to MI and sham, ∧*p* < 0.05 compared to sham; *n* = 5/group). One-way ANOVA followed by LSD *post hoc* test and independent samples *t*-test were used for statistical analysis (GFP^+^: CDCs *vs* sham *p* < 0.001, CDCs *vs* MI *p* < 0.001; GFP^−^: MI *vs* sham: *p* < 0.001, CDCs *vs* sham *p* < 0.001, CDCs *vs* MI *p* < 0.001; GFP^+^ *vs* GFP^−^ MI *p* = 0.001, GFP^+^ *vs* GFP^−^ CDCs *p* < 0.001; all other *p* = ns).**D.** Immunocytochemistry of enzymatically dissociated cardiomyocytes reveals that the percentage of GFP^+^ cardiomyocytes in the infarct border zone decreases post MI and is further decreased post CDC therapy, indicating contribution of endogenous progenitors to the myocyte pool (*p < 0.05 compared to sham, MI; *n* = 5/group). One-way ANOVA followed by LSD *post hoc* test was used for statistical analysis (CDCs *vs* sham *p* < 0.001; CDCs *vs* MI *p* = 0.023; all other *p* = ns). All error bars represent SDs. All scale bars: 20 µm.

Immunocytochemistry of dissociated cardiomyocytes for GFP, αSA and BrdU confirmed the results obtained by flow cytometry (Fig 8A–C) and showed a decrease in the frequency of GFP expression in BrdU^+^ cardiomyocytes after MI and after MI + CDC therapy (Supporting Information Fig 6), consistent with cardiomyogenic differentiation of recruited progenitors. Quantification of the percentages of GFP^+^ and GFP^−^ cardiomyocytes in the peri-infarct area by immunocytochemistry of enzymatically dissociated cells also revealed a dilution of the GFP^+^ pool by GFP^−^ cardiomyocytes after MI (71.2 ± 5.3% *vs* 79.2 ± 7.1% in sham), which became more pronounced after CDC therapy (61.4 ± 5.2%, *p* < 0.05 compared to sham, MI; Fig 8D), while tissue immunohistochemistry revealed higher rates of cardiomyocyte cycling in GFP^−^ cardiomyocytes compared to GFP^+^ cardioyocytes in the peri-infarct area after MI and after MI and CDC-therapy (Fig 5D, Supporting Information Fig 7). These results confirm that, in addition to proliferation of preformed cardiomyocytes, endogenous stem cells recruited post-MI replenish lost cardiomyocytes (Hsieh et al, 2007; Loffredo et al, 2011), and this phenomenon is amplified by CDC therapy.

### The magnitude of the observed phenomena cannot be explained by cardiomyocyte polyploidization, multinucleation, cell fusion or DNA repair

**Figure 9:**
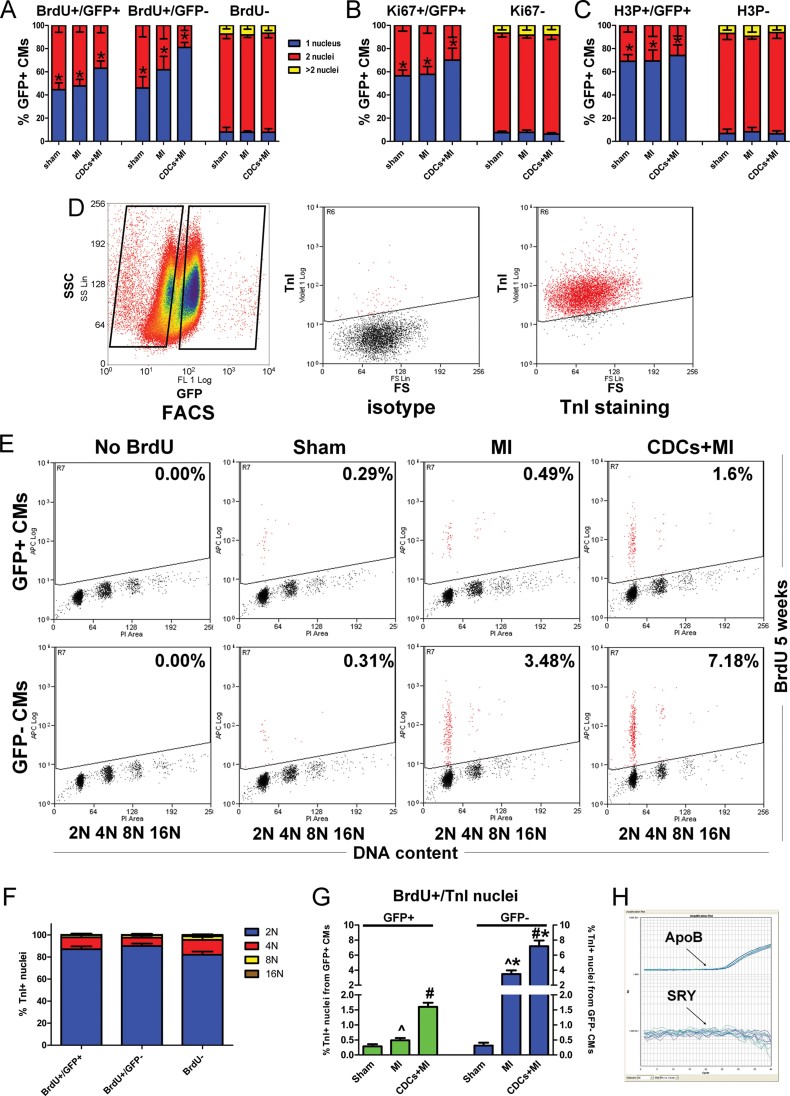
The magnitude of the observed DNA synthesis cannot be explained away by confounding factors **A–C.** Immunocytochemistry of enzymatically dissociated myocytes reveals that cycling (BrdU^+^, Ki67^+^, H3P^+^) GFP^+^ and GFP^−^ myocytes are more often mononucleated compared to non-cycling myocytes, thus excluding a prominent role of bi/multinucleation (*p < 0.05 compared to non-cycling cells, *n* = 4/group). One-way ANOVA followed by LSD *post hoc* test (**A**) and independent samples *t*-test (**B**,**C**) were used for statistical analysis (A: sham: BrdU^+^/GFP^+^ *vs* BrdU^−^ *p* < 0.001, BrdU^+^/GFP^−^ *vs* BrdU^−^ *p* < 0.001; MI: BrdU^+^/GFP^+^ *vs* BrdU^−^ *p* < 0.001, BrdU^+^/GFP^−^ *vs* BrdU^−^ *p* < 0.001; CDCs: BrdU^+^/GFP^+^ *vs* BrdU^−^ *p* < 0.001, BrdU^+^/GFP^−^ *vs* BrdU^−^ *p* < 0.001; B: sham: KI67^+^/GFP^+^ *vs* Ki67^−^ *p* < 0.001; MI: Ki67^+^/GFP^+^ *vs* Ki67^−^ *p* < 0.001; CDCs: Ki67^+^/GFP^+^ *vs* Ki67^−^ *p* = 0.001; C: sham: H3P^+^/GFP^+^ *vs* H3P^−^ *p* < 0.001; MI H3P^+^/GFP^+^ *vs* H3P^−^ *p* < 0.001; CDCs: H3P^+^/GFP^+^ vs H3P^−^ *p* < 0.001; all other *p* = ns).**D.** Nuclei isolated form FACS-sorted GFP^+^ and GFP^−^ cardiomyocytes were stained for TnI. Only TnI^+^ nuclei were examined.**E.** Flow cytometric analysis of isolated TnI^+^ nuclei from GFP^+^ and GFP^−^ FACS-sorted cardiomyocytes reveals that 87–90% of TnI^+^/BrdU^+^ nuclei are diploid, thus excluding a prominent role of bi/multinucleation (*n* = 4/group) Numbers in flow cytometry plots indicate averages for groups.**F.** Ploidy distributions did not differ significantly between BrdU^+^/TnI^+^ nuclei (obtained from GFP^+^ or GFP^−^ cardiomyocytes) and BrdU^−^/TnI^+^ nuclei (*n* = 4/group). One-way ANOVA followed by LSD *post hoc* test was used for statistical analysis.**G.** Percentages of BrdU^+^/TnI^+^ nuclei are similar to those obtained from flow cytometric analysis of whole cells (*p < 0.05 compared to nuclei from GFP^+^ cardiomyocytes; ^#^*p* < 0.05 compared to MI and sham, ∧*p* < 0.05 compared to sham; *n* = 4/group). One-way ANOVA followed by LSD *post hoc* test and independent samples *t*-test were used for statistical analysis (GFP^+^: MI *vs* sham: *p* = 0.018, CDCs *vs* sham *p* < 0.001, CDCs *vs* MI *p* < 0.001; GFP^−^: MI *vs* sham: *p* < 0.001, CDCs *vs* sham *p* < 0.001, CDCs *vs* MI *p* < 0.001; GFP^+^ *vs* GFP^−^ MI *p* < 0.001, GFP^+^ *vs* GFP^−^ CDCs *p* < 0.001; all other *p* = ns). All error bars represent SDs.**H.** DNA was extracted from GFP^+^ and GFP^−^ FACS-sorted cells obtained from female infarcted hearts injected with male CDCs. qPCR experiments using the male-specific SRY gene as a target resulted in no amplification, revealing no detectable fusion of exogenous CDCs with endogenous cardiomyocytes (*n* = 3).

DNA incorporation of nucleoside analogs or nuclear expression of cell-cycle proteins, while demonstrating cell-cycle activity, does not necessarily translate into genuine cell division and proliferation; potential confounding factors include polyploidization (DNA proliferation without karyokinesis and cytokinesis), bi/multinucleation (DNA proliferation and karyokinesis without cytokinesis), fusion of cardiomyocytes with non-cardiomyocyte cells, or DNA repair. In order to exclude a prominent role of polyploidization, nuclei isolated from GFP^+^ and GFP^−^ FACS-sorted cells (gated on large size) obtained from hearts of normal, infarcted and CDC-treated mice after 5 weeks of daily BrdU pulsing were analysed by flow cytometry for Troponin I (TnI) expression, BrdU incorporation and assessment of DNA content. We only examined TnI^+^ nuclei, to avoid contamination by non-myocyte nuclei (Bergmann et al, 2009, 2011; Fig 9D). We measured DNA content in isolated nuclei (rather than whole cells) in order to eliminate the confounding effects of different numbers of nuclei in myocytes (which, if analysing whole cells, would make it impossible to differentiate between mononucleated tetraploid myocytes and binucleated diploid myocytes). We found that 87 ± 2.6% of the BrdU^+^/TnI^+^ nuclei extracted from GFP^+^ cardiomyocytes, and 90 ± 2.2% of the BrdU^+^/TnI^+^ nuclei extracted from GFP^−^ cardiomyocytes were diploid (2N) (Fig 9E). The ploidy distributions (relative percentages of diploid, tetraploid [4N], octoploid [8N], and hexadecaploid [16N] nuclei) were comparable in BrdU^+^/TnI^+^ nuclei and BrdU^−^/TnI^+^ nuclei (Fig 9F). These findings exclude the possibility that the observed DNA synthesis can be explained by widespread cardiomyocyte polyploidization, as the latter would result in the majority of BrdU^+^/TnI^+^ nuclei being at least tetraploid (or octoploid/hexadecaploid) as well as in different ploidy distributions between BrdU^+^/TnI^+^ and BrdU^−^/TnI^+^ cardiomyocyte nuclei (with BrdU^+^ nuclei having higher DNA content). Based on our findings, 10–13% of BrdU^+^ cardiomyocyte nuclei were not diploid; it is unclear whether these nuclei represent instances where cell-cycle is activated abortively (replication of chromosomes without karyokinesis) or whether it occurs in newly formed myocytes that undergo another round of chromosomal replication. In any case, polyploidization can account for at most 10% (in GFP^−^ cardiomyocytes) to 13% (in GFP^+^ cardiomyocytes) of the observed DNA synthesis. The overall percentages of BrdU^+^/TnI^+^ nuclei in the sham, MI and CDC groups were similar to those obtained from flow cytometric analysis of whole cells (GFP^+^ fraction: sham = 0.29 ± 0.07%/5 weeks, MI = 0.49 ± 0.07%/5 weeks, CDCs = 1.6 ± 0.14%/5 weeks; GFP^−^ fraction: sham = 0.31 ± 0.10%/5 weeks, MI = 3.48 ± 0.49%/5 weeks, CDCs = 7.18 ± 0.77%/5 weeks; Fig 9G). We also compared ploidy distributions in nuclei isolated from GFP^+^ FACS-sorted cells obtained from 5-week-old and 1-year-old mice (that had been pulsed with 4-OH-Tamoxifen at 3 weeks of age). Taking into account that our results project to a resident cardiomyocyte turnover of ∼4% per year in the normal heart, a prominent role of polyploidization could translate into detectable differences in ploidy of isolated nuclei over time (as long as diploid and polyploid cardiomyocytes undergo apoptosis at equal rates). However, no differences in the relative percentages of diploid, tetraploid, octoploid and hexadecaploid nuclei could be observed between young and old mice (Supporting Information Fig 8A and B).

To exclude a prominent role of bi/multinucleation, we compared the relative percentages of mononucleated, binucleated and multinucleated myocytes in cycling GFP^+^, cycling GFP^−^ and non-cycling cardiomyocytes. Immynocytochemistry of isolated cells demonstrated that cycling [BrdU^+^ (measured after 5 weeks of pulsing), Ki67, H3P^+^ (measured 1 week after MI ± CDCs)] GFP^+^/αSA^+^ and cycling (BrdU^+^) GFP^−^/αSA^+^ cardiomyocytes were more often mononucleated compared to non-cycling (BrdU^−^, Ki67^−^, H3P^−^) αSA^+^ cardiomyocytes (Fig 9A–C), confirming previous reports (Chen et al, 2007). These findings exclude the possibility that the observed cell cycle activity can be explained exclusively by widespread cardiomyocyte bi/multinucleation. However, it should be noted that a substantial percentage of BrdU^+^ cardiomyocytes (GFP^+^ fraction: 55.5 ± 5.9% in the sham group, 52.3 ± 5.7% in the MI group and 37.0 ± 6.3% in the CDC group; GFP^−^ fraction: 54.0 ± 9.8% in the sham group, 38.3 ± 11.5% in the MI group and 19.1 ± 4.3% in the CDC group) were binucleated; it is unclear whether these myocytes represent instances where cell cycle is activated abortively (resulting in karyokinesis without cytokinesis), whether they represent newly formed mononucleated myocytes that completed another round of karyokinesis and became binucleated, or whether they represent progeny of dividing multinucleated cardiomyocytes [which have been shown to be capable of cytokinesis in vitro (Engel et al, 2005)]. We also compared the relative percentages of mononucleated, binucleated and multinucleated GFP^+^/αSA^+^ cells in 5-week-old and 1 year-old-mice (that had been pulsed with 4-OH-Tamoxifen at 3 weeks of age) by immunocytochemistry of isolated cardiomyocytes; no differences could be observed over time (Supporting Information Fig 8C), confirming previous reports (Olivetti et al, 1996).

To assess cell fusion, infarcted hearts of female bitransgenic mice were injected with male CDCs. DNA was extracted from GFP^+^ and GFP^−^ FACS-sorted cells (gated on large size) and qPCR for the male-specific SRY resulted in no amplification, revealing no detectable fusion of exogenous CDCs with cardiomyocytes [at least at a resolution of 1:40,000 cardiomyocytes which is the detection limit of qPCR (Zhang et al, 2010)] (Fig 9H). In principle, fusion phenomena could also occur between cardiomyocytes and other native host cell types; however, such phenomena are extremely rare (Alvarez-Dolado et al, 2003).

Finally, with regard to DNA damage and repair-associated BrdU incorporation, DNA repair leads to punctate labelling of nuclei by nucleoside analogs, appearing as isolated nuclear spots (reflecting integration at discrete sites of DNA damage) that cover <5% of the nuclear area (Kajstura et al, 2010); in our case, only cardiomyocytes with widespread, non-punctate labelling of the nucleus were considered BrdU^+^ (see Figs 3A, 4A,B and 8A,B). Moreover, DNA damage and repair cannot explain the detection of Ki67^+^ and H3P^+^ cardiomyocytes, as these proteins are not implicated in DNA repair (Scholzen & Gerdes, 2000).

Taken together, these results indicate that the magnitude of the observed DNA synthesis cannot be explained away by potential artefacts. At most, if all cases of polyploidization and bi/multinucleation are considered instances of abortive cell-cycle activation, these confounding factors can account for 68.5% (13%[polyploidization] + 55.5%[bi/multinucleation]), 65.3% (13 + 52.3%) and 50.0% (13 + 37%) of the measured DNA synthesis in GFP^+^ cardiomyocytes and for 64.0% (10%[polyploidization] + 54%[bi/multinucleation]), 48.3% (10 + 38.3%) and 29.1% (10 + 19.1%) of the measured DNA synthesis in GFP^−^ cardiomyocytes in the sham, MI and CDC treated mice, respectively.

### CDCs decrease scar size, increase viable myocardium and boost cardiac function after myocardial infarction

**Figure 10:**
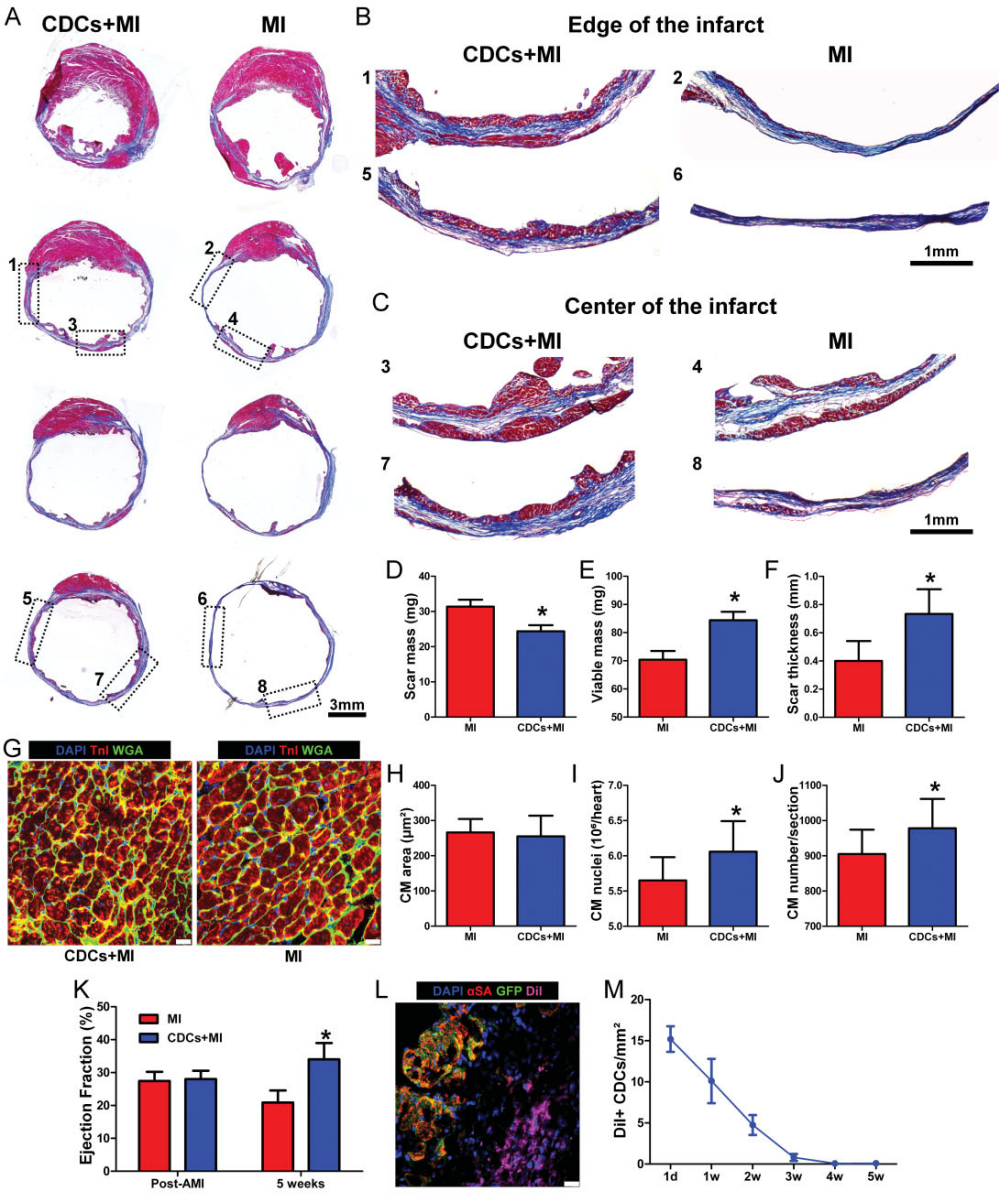
Structural, functional and regenerative benefits of CDC therapy **A–C.** Representative images of Masson's Trichrome-stained infarcted mouse hearts at 5 weeks post MI. Viable heart muscle stains red, while scar stains blue. Four consecutive sections per heart (obtained at 500 µm intervals, starting from the level of LAD ligation towards the apex) are presented. Note that the level of first section is similar, as manifested by the similar structure of the papillary muscles. Images in (**B**,**C**) are higher power images of the insets in (**A**), showing increased viable myocardium at the edges (**B**) and at the center (**C**) of the infarct in CDC-treated hearts.**D–F.** Morphometric analysis of CDC-treated and control hearts. CDC transplantation led to significant reduction of scar mass (*p* = 0.027) (**D**), increase of viable myocardium (*p* = 0.038) (**E**) and increased infarcted wall thickness (*p* = 0.005) (**F**), compared to control animals (*n* = 5–6/group).**G.** Representative images of the border zone revealing no myocyte hypertrophy in CDC-treated hearts.**H.** Border zone cardiomyocyte cross-sectional area did not differ significantly between groups (*n* = 10/group).**I.** Quantitative analysis of cardiomyocyte nuclei per heart (*n* = 10/group; *p* = 0.029).**J.** Quantitative analysis of cardiomyocytes per heart section (*n* = 10/group; *p* = 0.047).**K.** Echocardiographic assessment of LV function reveals that CDC transplantation resulted in superior global function (*p* < 0.001) compared to controls (*n* = 9/group).**L, M.** CDC engraftment as determined by immunohistochemistry after administration of DiI-labelled cells. Long-term cell survival beyond 3 weeks post-MI is low (*n* = 2/timepoint). All error bars represent SDs (*p < 0.05 compared to infarcted controls). All error bars represent SDs. Independent samples *t*-test was used for statistical analysis. Scale bars: 3 mm (**A**), 1 mm (**B**,**C**), 20 µm (**G**,**L**).

The increased incidence of cycling resident cardiomyocytes, and the increased cardiomyogenic differentiation of recruited progenitors after cell therapy, were accompanied by structural and functional benefits in the infarcted heart. Five weeks post-MI, CDC-treated hearts exhibited smaller scar mass, increased infarcted wall thickness and increased viable myocardium compared to controls (Fig 10A–F). To rule out myocyte hypertrophy as a contributor to the increase in viable myocardium [a phenomenon that has been reported after therapy with endothelial progenitor cells (Doyle et al, 2008)], we measured cardiomyocyte cross-sectional area in the peri-infarct area and found that cardiomyocyte size was similar in the CDC + MI and MI groups (Fig 10G and H). The cross-sectional area of cardiomyocytes located in the peri-infarct area (of both CDC-treated animals and infarcted controls) was higher than that of cardiomyocytes in remote myocardium; in addition, cardiomyocytes from both the peri-infarct and remote myocardium had increased area compared to cardiomyocytes in non-infarcted hearts (Supporting Information Fig 9). These data confirm previous reports demonstrating cardiomyocyte hypertrophy post-MI, which is more pronounced in the peri-infarct area compared to remote myocardium (Angeli et al, 2010; Lee et al, 2007). The fact that cardiomyocyte size in the peri-infarct area did not differ significantly between the CDC + MI and MI groups [even though CDC-treated hearts contained more BrdU^+^ cardiomyocytes in the peri-infarct area (Fig 5D, Supporting Information Fig 7) and cycling cardiomyocytes were smaller compared to non-cycling myocytes (Fig 4E)] can be rationalized by the relatively small percentage (∼4.7%) of additional cycling myocytes induced by CDC therapy in the peri-infarct area [which comprised ∼25% of the viable myocardium in our study (Supporting Information Fig 5)].

To unequivocally demonstrate that the increased viable myocardium in the CDC-treated hearts was a result of an increased number of myocytes, we directly counted cardiomyocyte nuclei and found that CDC-treated hearts had higher numbers of cardiomyocyte nuclei than did MI hearts (Fig 10I). We also compared cardiomyocyte numbers in heart sections (obtained at a fixed distance of 3 mm from the apex) and found increased numbers of myocytes in sections obtained from CDC-treated hearts compared to controls (Fig 10J). Importantly, global function was superior in CDC-treated animals compared to controls 5 weeks post-MI (Fig 10K), consistent with previous findings in small (Smith et al, 2007) and large animals (Lee et al, 2011). In addition, cardiac volumes were decreased in CDC-treated animals compared to infarcted controls (Supporting Information Fig 10), hinting towards attenuation of adverse remodelling. The observed beneficial effects occurred despite evanescent transplanted cell survival; minimal engraftment of CDCs (as assessed by immunohistochemistry after injection of DiI-labelled CDCs) was observed beyond the first 3 weeks post-MI (Fig 10L and M), confirming that the vast majority of the benefit is indirect (Chimenti et al, 2010; Malliaras et al, 2012). Proliferation and differentiation of transplanted cells appears to play a negligible role, consistent with the persistent functional and structural benefits of allogeneic CDC therapy long after the immunologically mismatched cells have been cleared (Malliaras et al, 2012).

## DISCUSSION

**Figure 11:**
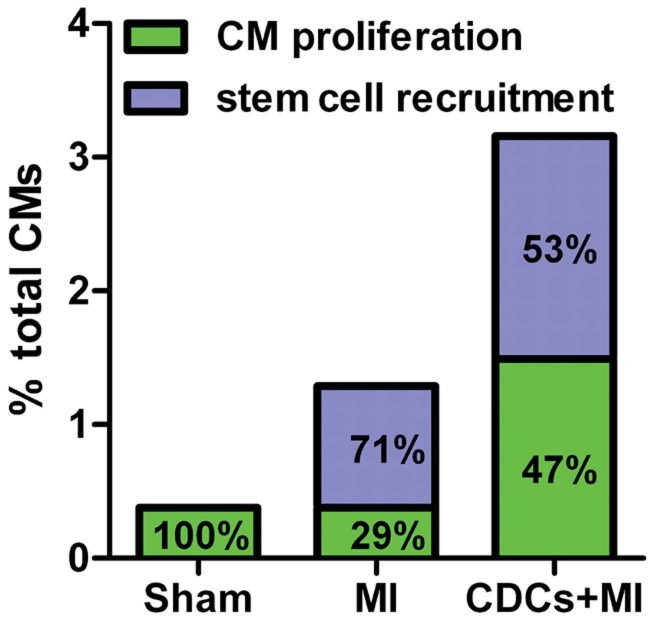
Origins of postnatal cardiomyogenesis in the normal, infarcted and cell-treated heart Both absolute rates (/5 weeks, y axis) and relative magnitudes (percentages inside bars) are presented (*n* = 5/group). In the normal mouse heart, cardiomyocyte turnover occurs through proliferation of resident cardiomyocytes. After MI, cardiomyocyte cycling increases, but the majority of new cardiomyocytes arise from recruited endogenous stem cells. Transplantation of CDCs upregulates host cardiomyocyte cycling and recruitment of endogenous progenitors.

We have attempted to determine the absolute rates and relative contributions of cardiomyocyte proliferation and cardiomyogenic differentiation of endogenous stem cells to myocyte replenishment in the normal, infarcted and cell-treated adult mammalian heart (Fig 11). Three aspects of our findings are notable. First, we demonstrate that myocyte replenishment occurs almost exclusively through proliferation of small mononucleated adult cardiomyocytes in the normal adult mouse heart, without any measurable contributions by endogenous progenitors [in agreement with some previous reports (Hsieh et al, 2007), but not others (Hosoda et al, 2009)]. We estimate an annual endogenous cardiomyocyte turnover of 1.3% (if we consider all cases of binucleation and polyploidization as instances where cell cycle is activated abortively) to 4% (if we consider all measured DNA synthesis as formation of new myocytes). The reported basal rate is comparable with that of some previous studies in mice (Soonpaa & Field, 1997) and humans (Bergmann et al, 2009), but contrasts starkly with other studies reporting either zero (Walsh et al, 2010) or very high rates of cardiomyogenesis (Kajstura et al, 2010) in the adult mammalian heart. The small but measurable level of resident cardiomyocyte cycling helps to rationalize the inability of the mammalian heart to undergo robust spontaneous regeneration, while raising hope that the endogenous regenerative potential could be amplified by therapeutic interventions. Second, we show that, after MI, cardiomyocyte cycling increases in the infarct border zone [confirming previous reports in mice (Hesse et al, 2012) and in humans (Beltrami et al, 2001)], but now differentiation of recruited stem cells becomes the dominant mechanism of cardiomyocyte replacement (in agreement with Hsieh et al, 2007 and Loffredo et al, 2011). Third, the most striking and surprising finding is the ability of cell therapy with CDCs, indirectly, to boost both cardiomyocyte proliferation as well as myocyte replenishment by recruited endogenous stem cells (Fig 11). This finding is in agreement with two recently published studies: one showing that cell therapy can indirectly increase the contribution of host progenitors to the myocyte pool (Loffredo et al, 2011) and another showing that therapeutic exploitation of the combination of enhanced stem cell recruitment and cardiomyocyte cell cycle induction results in increased myocardial renewal after MI (Zaruba et al, 2012). Two other studies have used the same fate mapping model to investigate postnatal cardiomyogenesis (Hsieh et al, 2007; Loffredo et al, 2011); however, they were designed primarily to detect contributions by endogenous progenitors to the myocyte pool, not proliferation of resident myocytes. In agreement with the previous studies, we find that endogenous progenitors do not replenish myocytes in the normal heart, but contribute to new myocyte formation post-MI (Hsieh et al, 2007), and this phenomenon can be amplified by cell therapy (Loffredo et al, 2011). The present work extends the previous studies by reporting that fate-mapped pre-existing resident cardiomyocytes contribute to new myocyte formation in the normal heart. We also demonstrate a substantial contribution of myocyte proliferation to the generation of new myocytes post-MI, a process that can be amplified after cell therapy with CDCs. The different results regarding the extent of resident cardiomyocyte proliferation in the normal, infarcted and cell-treated heart between our study and previous ones (Hsieh et al, 2007; Loffredo et al, 2011) can likely be attributed to: (a) the use of conventional histology in those studies to identify BrdU^+^ resident cardiomyocytes, a method that has been shown to be problematic for identification of cardiomyocyte nuclei (Ang et al, 2010); (b) the relatively short period of BrdU pulsing (1 week) in those studies, which decreases the likelihood of detecting rare events (like incorporation of BrdU into cardiomyocytes). Ultimately, newer techniques (Steinhauser et al, 2012) may provide additional insight into the relative contributions of the various cardiomyocyte replenishment mechanisms.

CDCs represent an attractive cell type for heart repair and regeneration. CDCs are clonogenic and exhibit multineage potential, thus fulfilling key criteria for stem cells (Davis et al, 2010). We and others have shown that CDCs improve cardiac function, reduce scar size and increase viable myocardium in small and large animals with ischemic cardiomyopathy (Johnston et al, 2009; Lee et al, 2011; Mishra et al, 2011; Smith et al, 2007). Importantly, CDCs are safe and effective in regenerating the infarcted human heart. In the prospective, randomized CADUCEUS trial, intracoronary infusion of autologous CDCs induced a significant decrease (∼12 g) in scar mass and a striking increase (∼22 g) in viable myocardium 1 year post therapy, while no significant changes were observed in the control group (Makkar et al, 2012). Nevertheless, the cellular origin of the newly formed myocardium remains unknown. Extensive preclinical evidence supports the idea that the mechanism of benefit is indirect: CDCs stimulate endogenous reparative/regenerative pathways, resulting in durable benefits despite ephemeral survival of the administered cells (Chimenti et al, 2010; Malliaras et al, 2012). Events of direct cardiomyogenic differentiation of CDCs *in vivo* represent “needles in the haystack” and do not contribute meaningfully to generation of new heart muscle (Malliaras et al, 2012). As a result, potential mechanisms to account for the increased viable myocardium after CDC therapy include host tissue preservation (protection of resident myocytes from apoptosis, modulation of inflammatory processes, increased angiogenesis), recruitment of endogenous progenitors and induction of resident cardiomyocyte proliferation (Malliaras & Marbán, 2011).

What are the relative contributions of these various mechanisms to the salutary effect of cell therapy with CDCs? Theoretical calculations (see Supporting Information) show that resident cardiomyocyte proliferation and cardiomyogenic differentiation of endogenous progenitors can account for a small fraction (7–19% and 16–21%, respectively) of the additional myocytes observed after CDC therapy; the majority of the increased viable myocardium in CDC-treated mice in the present acute MI model is likely the result of cytoprotection of pre-existing myocytes (Supporting Information Fig 11). In support of the latter, we found that CDC-treated hearts had reduced cardiomyocyte apoptosis (measured by TUNEL staining) compared to infarcted controls at 2 days post-MI (Supporting Information Fig 12), in agreement with previous reports showing reduced apoptosis post-CDC therapy (Li et al, 2012). New experiments in a chronic MI model will be required to eliminate tissue preservation from the equation, but the potency of this mechanism in acute MI gives good reason to explore the use of allogeneic CDCs as cardioprotective agents adjunctive to percutaneous coronary intervention.

Our study has several limitations. First, DNA incorporation of nucleoside analogs or nuclear expression of cell-cycle proteins, while demonstrating cell-cycle activity, does not necessarily translate into genuine cell division and proliferation. However, our results indicate that the magnitude of the measured DNA synthesis cannot be explained away by potential artefacts (polyploidization, bi/multinucleation, cell fusion or DNA repair) leaving the birth of new myocytes as the only likely explanation. Second, long-term BrdU pulsing may be toxic and affect the cycling rates of cardiomyocytes. Nevertheless, in our study no overt toxicity was observed after 5 weeks of daily BrdU administration, and the rates of actively cycling Ki67^+^ cardiomyocytes were similar in mice that received BrdU and in mice that did not receive BrdU. Third, it has been reported that cardiac-specific overexpression of GFP results in dilated cardiomyopathy when the levels of GFP expression are very high (Huang et al, 2000). However, we find that GFP^+^ and GFP^−^ cardiomyocytes exhibit similar rates of apoptosis (Supporting Information Fig 12) [confirming previous reports (Hsieh et al, 2007)] and no signs of cardiomyopathy were observed in sham-operated animals. Fourth, it has been reported that the α-myosin heavy chain promoter is active in c-Kit^+^ cells (Bailey et al, 2009). In our study, neither c-Kit^+^ nor Sca-1^+^ cells were detectable in GFP^+^ sorted cells (Fig 1C), verifying that the sorted cells are cardiomyocytes, not partially differentiated cardiac progenitor cells. In addition, no GFP^+^/c-Kit^+^ cells could be detected in mouse hearts (unpublished observation), confirming previous reports (Hsieh et al, 2007). However, we cannot exclude the possibility that some unknown progenitors were labelled by GFP after the 4-OH-Tamoxifen pulse. Fifth, our experimental design cannot determine whether the replicating cardiomyocytes arise from cell-cycle re-entry and proliferation of mature terminally differentiated myocytes, or from a specific subpopulation of myocytes that retain their ability to cycle and proliferate. The fact that cycling myocytes were predominantly small and mononucleated points towards the latter. Sixth, even though we perform theoretical calculations in order to determine the relative contributions of cardiomyocyte proliferation, differentiation of recruited endogenous stem cells and host tissue preservation to the salutary effect of cardiac cell therapy, it needs to be acknowledged that we did not perform a detailed evaluation of the time course of myocardial apoptosis post-MI. As a result, (even though we show reduced cardiomyocyte apoptosis at 2 days post-MI after CDC therapy) we cannot provide quantitative estimates of the absolute number of cardiomyocytes that are protected from apoptosis after therapy with CDCs.

Regardless of the precise percentages, we report the absolute rates and contributions of cardiomyocyte proliferation and cardiomyogenic differentiation of endogenous stem cells to myocyte replenishment in the normal, infarcted and cell-treated adult mammalian heart. Elucidation of the cellular sources of regenerative cardiomyogenesis is the first step towards development of novel therapeutic strategies that can improve the efficacy of stem cell-based treatments and bolster cardiomyocyte repopulation of infarcted myocardium.

## MATERIALS AND METHODS

### Generation of bitransgenic MerCreMer/ZEG Mice and 4-OH-Tamoxifen pulse

All animal procedures were approved by the Cedars-Sinai Medical Center Animal Care and Use Committee (IACUC2557). Bi-transgenic MerCreMer/ZEG mice were produced by crossbreeding cardiomyocyte-specific MerCreMer mice and ZEG mice (Jackson Laboratory). Animal genotype was verified by RT-PCR on tail genomic DNA (Zhang et al, 2010). Double heterozygous bitransgenic MerCreMer/ZEG mice were used for all experiments after induction of Cre recombination for GFP labelling in cardiomyocytes by 4-OH-Tamoxifen treatment. 4-OH-Tamoxifen (Sigma), dissolved in peanut oil (Sigma) at a concentration of 5 mg/ml, was intraperitoneally injected into 3–4-week-old MerCreMer/ZEG mice daily at a dosage of 0.5 mg/day. Fourteen consecutive injections of 4-OH Tamoxifen showed efficient and specific GFP labelling of cardiomyocytes.

### CDC culture and CM-DiI labelling

Mouse CDCs were expanded from explanted hearts obtained from 8-week-old male B6129SF1/J mice. The myocardial specimens were cut into fragments <1 mm^3^, washed and partially digested with trypsin (0.05%; GIBCO). These tissue fragments were culture as cardiac explants on fibronectin (20 mg/ml; Sigma) coated dishes in cardiac explant media [CEM; Iscove's Modified Dulbecco's Medium (GIBCO), foetal bovine serum 20% (HyClone, Logan, UT), 100 U/ml penicillin G (GIBCO), 100 U/ml streptomycin (GIBCO) and 0.1 mmol/L 2-mercaptoethanol (GIBCO)]. After a variable period of growth, a layer of stromal-like cells emerged from the cardiac explant over which phase bright cells proliferated. The loosely adherent cells surrounding the explant (termed cardiac outgrowth) were harvested using mild enzymatic digestion (0.05% trypsin under direct visualization, GIBCO). Cardiac outgrowth could be harvested up to four more times from the same specimen. Harvested cardiac outgrowth was seeded at 50,000 cells/ml on poly-d-lysine coated dishes in CEM. Several days later, cells that remained adherent to the poly-d-lysine coated dishes were discarded, while free-floating cardiospheres were harvested, plated on fibronectin coated flasks and cultured in CEM to generate CDCs. In order to quantify engraftment, in a subset of experiments CM-DiI (Molecular probes) labelled CDCs were used.

### Myocardial infarction creation, CDC injection and BrdU pulse

An acute MI was created in young adult bitransgenic MerCreMer/ZEG mice (6–8 weeks old). After general anaesthesia and tracheal intubation, mice were artificially ventilated with room air. A left thoracotomy was performed through the fourth intercostal space and the left anterior descending artery was ligated with 9-0 prolene under direct vision. Mice were then subjected to intramyocardial injections with a 30 G needle at four points in the infarct border zone with 40 µl of PBS (MI group) or 2 × 10^5^ CDCs (CDCs + MI group). Mice in the sham group underwent left thoracotomy without LAD ligation. After injections were completed, the chest was closed, anaesthesia was discontinued and the animals were allowed to recover. In order to monitor proliferation of endogenous cardiomyocytes, animals were intraperitoneally injected with BrdU (100 mg/kg body weight) once daily for up to 5 weeks post MI. In a subset of experiments (Supporting Information Fig 3), mice did not receive any BrdU administration.

**The paper explained**

PROBLEM:

Myocardial infarction kills living heart muscle, leading to its replacement by scar. Cell therapy with cardiosphere-derived cells has been shown to be clinically effective in regenerating the infarcted human heart. However, the cellular origin of the newly formed myocardium (proliferation of pre-existing cardiomyocytes *vs* differentiation of endogenous progenitors) remains unknown.

RESULTS:

We demonstrate that the normal adult mouse heart replenishes lost myocytes almost exclusively through proliferation of pre-existing cardiomyocytes, without any measurable contributions by endogenous progenitors. After myocardial infarction, proliferation of resident myocytes increases, but the majority of new cardiomyocytes arise from recruited endogenous stem cells. Cell therapy with cardiosphere-derived cells boosts both adult cardiomyocyte proliferation as well as myocyte replenishment by recruited endogenous stem cells, resulting in the growth of new healthy heart muscle.

IMPACT:

Elucidation of the cellular sources of regenerative cardiomyogenesis is the first step towards development of novel therapeutic strategies that can improve the efficacy of stem cell-based treatments and bolster cardiomyocyte repopulation of infarcted myocardium.

### Enzymatic dissociation of explanted hearts

Cardiomyocytes were isolated from MerCreMer/ZEG mice by enzymatic dissociation of the whole heart on a Langendorff apparatus. Heparinized animals were anaesthetized by Ketamine/Xylazine (30 and 6 mg/Kg, respectively). Hearts were rapidly excised and cleansed to remove blood in ice-cold Tyrode's solution before being mounted to a Langendorff apparatus conjugated to a pressure monitoring device, then perfused retrogradely with the following two oxygenated solutions in sequential order: 1. Ca^2+^-free Tyrode's solution (2–3 min). Ca^2+^-free Tyrode's solution containing 1 mg/ml collagenase B (Roche) and 0.1 mg/ml protease (Sigma) for 10–15 min depending on digesting conditions. Hearts were subsequently dismounted, atria were discarded and digested ventricles were cut off and minced in Kruftbrühe (KB) solution, followed by pipetting to dissociate the cells and filtered through a nylon mesh (250 µm pore size) to remove big pieces of undigested tissues. Isolated cells were rinsed in KB solution and allowed to settle by gravity to remove debris and non-cardiomyocytes. The remaining cells (comprising both cardiomyocytes and other cells) were then resuspended in KB solution for further experiments. Tyrode's solution contained (mmol/L): NaCl 105, KCl 5.4, KH_2_PO_4_ 0.6, NaH_2_PO_4_ 0.6, NaHCO_3_ 6, KHCO_3_ 5, MgC_l2_ 1, HEPES 10, glucose 5, taurine 20, BDM 20 (pH 7.4 with NaOH). KB solution contained (mmol/L): KCl 20, KH_2_PO_4_ 10, K^+^-glutamate 70, MgCl_2_ 1, glucose 25, *b*-hydroxybutyric acid 10, taurine 20, EGTA 0.5, HEPES 10 and 0.1% albumin (pH 7.4 with KOH). Chemicals were purchased from Sigma.

### *Ex vivo* perfusion of hearts with fluorescent dye

Hearts were mounted to a Langendorff apparatus as described above, then perfused retrogradely with oxygenated Tyrode's solution containing 6 µM Celltracker RED (Molecular probes/Invitrogen) for 30 min. Tyrode's solution contained (mmol/L): NaCl 135, KCl 5, CaCl_2_ 1.8, MgCl_2_ 1.2, HEPES 10, glucose 10 (pH 7.4 with NaOH). Afterwards, hearts were enzymatically dissociated as described above.

### FACS sorting of cardiomyocytes

Enzymatically dispersed cell preparations resuspended in KB solution were sorted on a MoFlo Cell Sorter (DakoCytomation, Inc.) with a flow rate of 500 cells/s, using a 150 µm nozzle (Diez & Simm, 1998). Cardiomyocytes were sorted based on cell size (high forward and side scatter) and GFP expression. Non-Tamoxifen pulsed bitransgenic mice were used as controls.

### Flow cytometry

Flow cytometry experiments were performed in order to measure nuclear incorporation of BrdU and expression of Ki67 in sorted GFP^+^ cardiomyocytes using commercially available kits (BD Pharmingen), according to manufacturer's instructions. Flow cytometry experiments were performed in order to measure nuclear incorporation of BrdU (using commercially available kits [BD Pharmingen]) and expression of αSA (Abcam) in sorted cardiomyocytes. In addition, general phenotypic characterization of sorted GFP^+^ cardiomyocytes (expression of GFP, Sca-1, c-Kit, CD90, CD31 and αSMA) was performed. Flow cytometry was also performed in nuclei isolated from sorted GFP^+^ cardiomyocytes in order to determine BrdU incorporation (using commercially available kits [BD pharmingen]), DNA content (Propidium Iodide staining) and TnI (Abcam) expression. Nuclei were isolated from GFP^+^-sorted cells using a commercially available nuclei isolation kit (Sigma), according to the manufacturer's instructions. Flow cytometry experiments were performed using a benchtop flow cytometer (CyAnADP; DakoCytomation, Inc.). Gates were established by forward and side scatter to exclude cellular debris. Fluorescent compensation was performed using single labelled controls. The percentage of positive cells was defined as the percent of the population falling 99.7% of an isotype-matched antibody control cell population. Quantitative analysis was performed using Summit software (Beckman Coulter, Inc.).

### PCR microarray analysis

Using the RT2 Profiler PCR Array System (SABiosciences), we compared expression of cell-cycle associated genes in RNA extracted from GFP^+^ sorted cardiomyocytes using the ΔΔ*C*_t_ method. Total RNA was extracted from GFP^+^ cells with RNeasy Pus Micro kit (Qiagen) according to the manufacturer's instructions. cDNA was prepared from the total RNA using the RT2 First Strand Kit (SABiosiences). Experimental cocktail was prepared by adding cDNA to RT2 qPCR Master Mix (SABiosiences) within the 96-well PCR array. Real-time PCR was performed in 7900HT Fast real-time PCR System. Data were analysed using the 7900HT Sequence Detection System Software (Applied Biosystems) and PCR Array Data Analysis Software (SABiosiences).

### Immunocytochemistry of enzymatically dissociated cardiomyocyte preparations

Enzymatically dissociated myocyte preparations were cytospun on to laminin-coated slides. Fluorescent immunocytochemistry was performed with antibodies against α-sarcomeric actinin (Sigma), GFP (Abcam), Ki67 (Thermo Scientific), BrdU (Roche), H3P (Abcam) and lacZ (Abcam).

### Histology

Hearts were arrested with KCl solution, explanted, frozen in OCT compound, and sectioned in 6 or 10 µm sections on a cryostat. Cryosections were subsequently fixed with 4% paraformaldehyde. Quantitative morphometric analysis with Masson's trichrome staining was performed on 6 µm sections (four sections per heart at 500 µm intervals, starting at the level of LAD ligation). Scar and viable mass was measured by multiplying the scar and viable volume with the specific gravity of myocardium (1.05 g/cm^3^). Peri-infarct area was defined as the area within one low-power field (10×) from the edges of the scar, but not including the scar (Supporting Information Fig 5). Cardiomyocyte nuclei were counted using the optical dissector method on Troponin I and DAPI-stained 10 µm sections in five random sample volumes per heart (Howard & Reed, 2005). Total number of cardiomyoctes per section was quantified in 6 µm sections obtained at 3 mm from the apex. Sections were stained with Troponin I (Abcam), α-sarcomeric actinin (Sigma), GFP (Abcam), Ki67 (Thermo Scientific), BrdU (Roche), lacZ (Abcam), WGA (Molecular probes), isolectin (Molecular probes), Connexin 43 (Sigma), TUNEL staining (Roche). In all immunocytochemistry and immunohistochemistry experiments, Alexa Fluor conjugated secondary antibodies (Molecular probes) were used and counterstaining with DAPI (Molecular probes) was performed. Sections were imaged using a confocal laser scan microscope (Leica Microsystems) and images were processed by Leica LAS software suite.

### PCR to exclude fusion of exogenous CDCs with endogenous cardiomyocytes

In order to exclude fusion of exogenous CDCs with endogenous cardiomyocytes, a subset of female bitransgenic MErCreMer/ZEG mice were injected with male CDCs. GFP^+^ and GFP^−^ cardiomyocytes were sorted as described and DNA was extracted from the sorted cells with the DNeasy Blood & Tissue Kit (Qiagen). We then investigated fusion between male CDCs and female cardiomyocytes by real-time PCR using the mouse SRY gene located on the Y chromosome as target, using commercially available primers and probes (Applied Biosystems). ApoB was used as an endogenous control. For each reaction, 50 ng of template DNA was used. Real-time PCR was performed in 7900HT Fast real-time PCR System.

### Echocardiography

Mice underwent echocardiography at 5 h (baseline) and 5 weeks after surgery using Vevo 770TM Imaging System (VISUALSONICS). After the induction of light general anaesthesia, the hearts were imaged two-dimensionally (2D) in long-axis views at the level of the greatest LV diameter. LV ejection fraction (LVEF), LV end-diastolic volume and LV end-systolic volume were measured with VisualSonics V1.3.8 software from 2D long-axis views taken through the infarcted area.

### Statistics

Results both in text and in figures are presented as means ± SD. Statistical significance was determined by independent samples *t*-test and one-way ANOVA followed by LSD *post hoc* test, or Dunnett's *post hoc* test (SPSS 16.0). Differences were considered significant when *p* < 0.05.

## Author contributions

KM established the hypotheses, designed the study, performed the experiments, analysed data and wrote the paper; YZ was involved in study design and co-analysed data; JS performed histology and assisted with enzymatical dissociation of hearts; GG performed histology; ET assisted with enzymatical dissociation of hearts and Tamoxifen/BrdU pulsing of mice; KC performed confocal imaging; BS performed the mouse surgeries; MA cultured CDCs; EM established the hypotheses, designed the study, co-analysed data and wrote the paper.

## Supplementary material

As a service to our authors and readers, this journal provides supporting information supplied by the authors. Such materials are peer reviewed and may be re-organized for online delivery, but are not copy-edited or typeset. Technical support issues arising from supporting information (other than missing files) should be addressed to the authors.

## References

[b1] Ang KL, Shenje LT, Reuter S, Soonpaa MH, Rubart M, Field LJ, Galiñanes M (2010). Limitations of conventional approaches to identify myocyte nuclei in histologic sections of the heart. Am J Physiol Cell Physiol.

[b2] Angeli FS, Amabile N, Burjonroppa S, Shapiro M, Bartlett L, Zhang Y, Virmani R, Chatterjee K, Boyle A, Grossman W (2010). Prolonged therapy with erythropoietin is safe and prevents deterioration of left ventricular systolic function in a porcine model of myocardial infarction. J Card Fail.

[b3] Alvarez-Dolado M, Pardal R, Garcia-Verdugo JM, Fike JR, Lee HO, Pfeffer K, Lois C, Morrison SJ, Alvarez-Buylla A (2003). Fusion of bone-marrow-derived cells with Purkinje neurons, cardiomyocytes and hepatocytes. Nature.

[b4] Bailey B, Izarra A, Alvarez R, Fischer KM, Cottage CT, Quijada P, Díez-Juan A, Sussman MA (2009). Cardiac stem cell genetic engineering using the alphaMHC promoter. Regen Med.

[b5] Beltrami AP, Urbanek K, Kajstura J, Yan SM, Finato N, Bussani R, Nadal-Ginard B, Silvestri F, Leri A (2001). Evidence that human cardiac myocytes divide after myocardial infarction. N Engl J Med.

[b6] Bergmann O, Bhardwaj RD, Bernard S, Zdunek S, Barnabé-Heider F, Walsh S, Zupicich J, Alkass K, Buchholz BA, Druid H (2009). Evidence for cardiomyocyte renewal in humans. Science.

[b7] Bergmann O, Zdunek S, Alkass K, Druid H, Bernard S, Frisén J (2011). Identification of cardiomyocyte nuclei and assessment of ploidy for the analysis of cell turnover. Exp Cell Res.

[b8] Boström P, Mann N, Wu J, Quintero PA, Plovie ER, Panáková D, Gupta RK, Xiao C, MacRae CA, Rosenzweig A (2010). C/EBPβ controls exercise induced cardiac growth and protects against pathological cardiac remodeling. Cell.

[b9] Chen X, Wilson RM, Kubo H, Berretta RM, Harris DM, Zhang X, Jaleel N, MacDonnell SM, Bearzi C, Tillmanns J (2007). Adolescent feline heart contains a population of small, proliferative ventricular myocytes with immature physiological properties. Circ Res.

[b10] Chimenti I, Smith RR, Li TS, Gerstenblith G, Messina E, Giacomello A, Marbán E (2010). Relative roles of direct regeneration versus paracrine effects of human cardiosphere-derived cells transplanted into infarcted mice. Circ Res.

[b11] Davis DR, Smith RR, Marbán E (2010). Human cardiospheres are a source of stem cells with cardiomyogenic potential. Stem Cells.

[b12] Diez C, Simm A (1998). Gene expression in rod shaped cardiac myocytes, sorted by flow cytometry. Cardiovasc Res.

[b13] Doyle B, Sorajja P, Hynes B, Kumar AH, Araoz PA, Stalboerger PG, Miller D, Reed C, Schmeckpeper J, Wang S (2008). Progenitor cell therapy in a porcine acute myocardial infarction model induces cardiac hypertrophy, mediated by paracrine secretion of cardiotrophic factors including TGFbeta1. Stem Cells Dev.

[b14] Engel FB, Schebesta M, Duong MT, Lu G, Ren S, Madwed JB, Jiang H, Wang Y, Keating MT (2005). p38 MAP kinase inhibition enables proliferation of adult mammalian cardiomyocytes. Genes Dev.

[b15] Hatzistergos KE, Quevedo H, Oskouei BN, Hu Q, Feigenbaum GS, Margitich IS, Mazhari R, Boyle AJ, Zambrano JP, Rodriguez JE (2010). Bone marrow mesenchymal stem cells stimulate cardiac stem cell proliferation and differentiation. Circ Res.

[b16] Hesse M, Raulf A, Pilz GA, Haberlandt C, Klein AM, Jabs R, Zaehres H, Fügemann CJ, Zimmermann K, Trebicka J (2012). Direct visualization of cell division using high-resolution imaging of M-phase of the cell cycle. Nat Commun.

[b17] Hosoda T, D'Amario D, Cabral-Da-Silva MC, Zheng H, Padin-Iruegas ME, Ogorek B, Ferreira-Martins J, Yasuzawa-Amano S, Amano K, Ide-Iwata N (2009). Clonality of mouse and human cardiomyogenesis in vivo. Proc Natl Acad Sci USA.

[b18] Howard CV, Reed MG, Jones C (2005). Unbiased stereology: Three-dimensional measurement. Microscopy.

[b19] Hsieh PC, Segers VF, Davis ME, MacGillivray C, Gannon J, Molkentin JD, Robbins J, Lee RT (2007). Evidence from a genetic fate-mapping study that stem cells refresh adult mammalian cardiomyocytes after injury. Nat Med.

[b20] Huang WY, Aramburu J, Douglas PS, Izumo S (2000). Transgenic expression of green fluorescence protein can cause dilated cardiomyopathy. Nat Med.

[b21] Jecker P, Beuleke A, Dressendörfer I, Pabst R, Westermann J (1997). Long-term oral application of 5-bromo-2-deoxyuridine does not reliably label proliferating immune cells in the LEW rat. J Histochem Cytochem.

[b22] Johnston PV, Sasano T, Mills K, Evers R, Lee ST, Smith RR, Lardo AC, Lai S, Steenbergen C, Gerstenblith G (2009). Engraftment, differentiation, and functional benefits of autologous cardiosphere-derived cells in porcine ischemic cardiomyopathy. Circulation.

[b23] Kajstura J, Urbanek K, Perl S, Hosoda T, Zheng H, Ogórek B, Ferreira-Martins J, Goichberg P, Rondon-Clavo C, Sanada F (2010). Cardiomyogenesis in the adult human heart. Circ Res.

[b24] Kikuchi K, Holdway JE, Werdich AA, Anderson RM, Fang Y, Egnaczyk GF, Evans T, Macrae CA, Stainier DY, Poss KD (2010). Primary contribution to zebrafish heart regeneration by gata4+ cardiomyocytes. Nature.

[b25] Kimbrough A, Kwon B, Eckel LA, Houpt TA (2011). Systemic 5-bromo-2 deoxyuridine induces conditioned flavor aversion and c-Fos in the visceralneuraxis. Learn Mem.

[b26] Laflamme MA, Murry CE (2011). Heart regeneration. Nature.

[b27] Lee ST, White AJ, Matsushita S, Malliaras K, Steenbergen C, Zhang Y, Li TS, Terrovitis J, Yee K, Simsir S (2011). Intramyocardial injection of autologous cardiospheres or cardiosphere-derived cells preserves function and minimizes adverse ventricular remodeling in pigs with heart failure post-myocardial infarction. J Am Coll Cardiol.

[b28] Lee TM, Lin MS, Chang NC (2007). Inhibition of histone deacetylase on ventricular remodeling in infarcted rats. Am J Physiol Heart Circ Physiol.

[b29] Li JM, Brooks G (1999). Cell cycle regulatory molecules (cyclins, cyclin-dependent kinases and cyclin-dependent kinase inhibitors) and the cardiovascular system; potential targets for therapy. Eur Heart J.

[b30] Li TS, Cheng K, Malliaras K, Smith RR, Zhang Y, Sun B, Matsushita N, Blusztajn A, Terrovitis J, Kusuoka H (2012). Direct comparison of different stem cell types and subpopulations reveals superior paracrine potency and myocardial repair efficacy with cardiosphere-derived cells. J Am Coll Cardiol.

[b31] Loffredo FS, Steinhauser ML, Gannon J, Lee RT (2011). Bone marrow-derived cell therapy stimulates endogenous cardiomyocyte progenitors and promotes cardiac repair. Cell Stem Cell.

[b32] Makkar RR, Smith RR, Cheng K, Malliaras K, Thomson L, Berman D, Czer L, Marbán L, Mendizabal A, Johnston PV (2012). Heart regeneration after myocardial infarction in patients treated with intracoronary cardiosphere-derived cells: results of a first-in-human prospective, randomised trial. Lancet.

[b33] Malliaras K, Marbán E (2011). Cardiac cell therapy: where we've been, where we are, and where we should be headed. Br Med Bull.

[b34] Malliaras K, Li TS, Luthringer D, Terrovitis J, Cheng K, Chakravarty T, Galang G, Zhang Y, Schoenhoff F, Van Eyk J (2012). Safety and efficacy of allogeneic cell therapy in infarcted rats transplanted with mismatched cardiosphere-derived cells. Circulation.

[b35] Mishra R, Vijayan K, Colletti EJ, Harrington DA, Matthiesen TS, Simpson D, Goh SK, Walker BL, Almeida-Porada G, Wang D (2011). Characterization and functionality of cardiac progenitor cells in congenital heart patients. Circulation.

[b36] Olivetti G, Cigola E, Maestri R, Corradi D, Lagrasta C, Gambert SR, Anversa P (1996). Aging, cardiac hypertrophy and ischemic cardiomyopathy do not affect the proportion of mononucleated and multinucleated myocytes in the human heart. J Mol Cell Cardiol.

[b37] Pasumarthi KB, Field LJ (2002). Cardiomyocyte cell cycle regulation. Circ Res.

[b38] Porrello ER, Mahmoud AI, Simpson E, Hill JA, Richardson JA, Olson EN, Sadek HA (2011). Transient regenerative potential of the neonatal mouse heart. Science.

[b39] Scholzen T, Gerdes J (2000). The Ki-67 protein: from the known and the unknown. J Cell Physiol.

[b40] Smith RR, Barile L, Cho HC, Leppo MK, Hare JM, Messina E, Giacomello A, Abraham MR, Marbán E (2007). Regenerative potential of cardiosphere-derived cells expanded from percutaneous endomyocardial biopsy specimens. Circulation.

[b41] Soonpaa MH, Field LJ (1997). Assessment of cardiomyocyte DNA synthesis in normal and injured adult mouse hearts. Am J Physiol.

[b42] Steinhauser ML, Lee RT (2011). Regeneration of the heart. EMBO Mol Med.

[b43] Steinhauser ML, Bailey AP, Senyo SE, Guillermier C, Perlstein TS, Gould AP, Lee RT, Lechene CP (2012). Multi-isotope imaging mass spectrometry quantifies stem cell division and metabolism. Nature.

[b44] Walsh S, Pontén A, Fleischmann BK, Jovinge S (2010). Cardiomyocyte cell cycle control and growth estimation in vivo – an analysis based on cardiomyocyte nuclei. Cardiovasc Res.

[b45] Weghorst CM, Henneman JR, Ward JM (1991). Dose response of hepatic and renal DNA synthetic rates to continuous exposure of bromodeoxyuridine (BrdU) via slow-release pellets or osmotic minipumps in male B6C3F1 mice. J Histochem Cytochem.

[b46] Zaruba MM, Zhu W, Soonpaa MH, Reuter S, Franz WM, Field LJ (2012). Granulocyte colony-stimulating factor treatment plus dipeptidylpeptidase-IV inhibition augments myocardial regeneration in mice expressing cyclin D2 in adult cardiomyocytes. Eur Heart J.

[b47] Zhang Y, Li TS, Lee ST, Wawrowsky KA, Cheng K, Galang G, Malliaras K, Abraham MR, Wang C, Marbán E (2010). Dedifferentiation and proliferation of mammalian cardiomyocytes. PLoS One.

