# Supplementary material for: Cardiomyocyte proliferation and progenitor cell recruitment underlie therapeutic regeneration after myocardial infarction in the adult mouse heart
Source: EMBO Mol Med. 2013 Jan 29;5(2):191–209. doi: 10.1002/emmm.201201737 (PMC3569637; doi:10.1002/emmm.201201737)
Supplement: Supplementary file 1 [file emmm0005-0191-SD1.pdf]

EMBO Molecular Medicine

Cardiomyocyte proliferation and progenitor cell recruitment underlie therapeutic regeneration after myocardial infarction in the adult mouse heart

Konstantinos Malliaras, Yiqiang Zhang, Jeffrey Seinfeld, Giselle Galang, Eleni Tseliou, Ke Cheng, Baiming Sun, Mohammad Aminzadeh, Eduardo Marbán

Corresponding author: Eduardo Marbán, Cedars-Sinai Heart Institute

Review timeline:

Submission date:	13 July 2012
Accepted:	07 December 2012

Transaction Report:

No Peer Review Process File is available with this article, as the authors have chosen not to make the review process public in this case.

© EMBO
