# Supplementary material for: Cardiomyocyte proliferation and progenitor cell recruitment underlie therapeutic regeneration after myocardial infarction in the adult mouse heart
Source: EMBO Mol Med. 2013 Jan 29;5(2):191–209. doi: 10.1002/emmm.201201737 (PMC3569637; doi:10.1002/emmm.201201737)
Supplement: Supplementary file 2 [file emmm0005-0191-SD2.pdf]

Supporting Information

Table of contents

Calculation of the relative contributions of myocyte proliferation, stem cell differentiation and cardioprotection (page 2)

Supporting Information Fig 1 (page 9)

Supporting Information Fig 2 (page 10)

Supporting Information Fig 3 (page 12)

Supporting Information Fig 4 (page 13)

Supporting Information Fig 5 (page 14)

Supporting Information Fig 6 (page 15)

Supporting Information Fig 7 (page 16)

Supporting Information Fig 8 (page 17)

Supporting Information Fig 9 (page 18)

Supporting Information Fig 10 (page 19)

Supporting Information Fig 11 (page 20)

Supporting Information Fig 12 (page 21)

Calculation of the relative contributions of myocyte proliferation, stem cell differentiation and cardioprotection

We attempted to calculate the relative contributions of myocyte proliferation, stem cell differentiation and cardioprotection to the salutary effect of cell therapy with CDCs with 4 different methods. First, we directly counted cardiomyocyte nuclei in infarcted hearts; 5 weeks post MI, CDC-treated hearts had 410000 more cardiomyocyte nuclei compared to controls (6.06×10^6 vs 5.65×10^6 nuclei). Flow cytometric analysis of isolated TnI+ nuclei extracted from FACS-sorted endogenous GFP+ cardiomyocytes demonstrated that 1.6% of resident cardiomyocyte TnI+ nuclei were BrdU+ 5 weeks after CDC treatment, compared to 0.49% in infarcted controls. As a result, CDC-induced resident cardiomyocyte cycling resulted in generation of 69275 additional cardiomyocyte nuclei ($1.6\% \times 6.06 \times 10^6 - 0.49\% \times 5.65 \times 10^6$), equivalent to 17% of the observed effect (69275/410000). Flow cytometric analysis of isolated TnI+ nuclei extracted from FACS-sorted GFP- cardiomyocytes demonstrated that 7.2% (an additional 5.6% compared to the GFP+ fraction) of TnI+ nuclei were BrdU+ 5 weeks after CDC treatment, compared to 3.5% (an additional 3.01% compared to the GFP+ fraction) in infarcted controls. As a result (and taking into account that GFP- myocytes were 29% and 39% of total border zone myocytes after MI and MI+CDCs respectively), CDC-induced cardiomyogenic differentiation of recruited progenitors resulted in generation of 74032 additional cardiomyocyte nuclei ($5.6\% \times 39\% \times 6.06 \times 10^6 - 3.01\% \times 29\% \times 5.65 \times 10^6$), equivalent to 18% of the observed effect (74032/410000). The rest of the effect (100%-17%-18%=65% of the additional nuclei observed after therapy with CDCs) should be attributable to host

tissue preservation leading to more salvaged myocardium. Using the approach described above, the relative contributions of cardiomyocyte proliferation versus cardiomyogenic differentiation of host stem cells in myocyte replenishment post-MI were 19% and 81% respectively; after CDC therapy, the relative contributions to regenerative cardiomyogenesis were 48% (for cardiomyocyte proliferation and 52% for differentiation of progenitors) . Thus, CDC therapy disproportionately upregulates cardiomyocyte proliferation, although it also increases stem cell recruitment to a lesser degree.

Second, we counted the total number of cardiomyocytes in heart sections obtained at a specific distance from the apex. We found that CDC-treated hearts had on average 8% more cardiomyocytes per section compared to infarcted controls 5 weeks post-MI (978 vs 905 cardiomyocytes/section). Taking into account that (according to the flow cytometry results) 2.08% of resident GFP+ cardiomyocytes were BrdU+ at 5 weeks compared to 0.76% in controls, we then assumed 2 scenarios: a) if all observed DNA synthesis translated to resident cardiomyocyte proliferation, CDC-induced cardiomyocyte cycling would result in generation of 2.08x1.08% (since CDC treated hearts had 8% more myocytes) – 0.76% = 1.49% more myocytes in CDC treated hearts compared to controls; this is equivalent to 19% of the observed effect (1.49%/8%); b) if all cases of binucleation/ polyploidization represent instances where cell-cycle is activated abortively in resident cardiomyocytes, then only 34.7% of the observed DNA synthesis in controls (100% - 13% [polyploidization] – 52.3% [binucleation]) and 50% in CDC-treated animals (100% - 13% [polyploidization] – 37% [binucleation]) should be attributed to new myocyte generation. In this case, CDC-induced cardiomyocyte cycling would result in generation of 2.08x50%x1.08% – 0.76%x34.7% = 0.88% more myocytes in CDC-

treated hearts compared to controls; this is equivalent to 11% of the observed effect (0.86%/8%). We followed the same approach to quantify the contribution of stem cells to the myocyte pool. Flow cytometric analysis in GFP- cardiomyocytes demonstrated that 8.2% (an additional 6.1% compared to the GFP+ fraction) of GFP-/áSA+ cardiomyocytes were BrdU+ 5 weeks after CDC treatment, compared to 3.9% (an additional 3.1% compared to the GFP+ fraction) in infarcted controls. In addition, GFP- myocytes accounted for 29% and 39% of total border zone myocytes after MI and MI+CDCs respectively. Based on the above, if all observed DNA synthesis translated new myocyte formation, CDC-induced host stem cell myocyte replenishment re-entry would account for generation of 6.1%x39%x1.08% (since CDC treated hearts had 8% more myocytes) – 3.1%x29% = 1.67% more myocytes in CDC treated hearts compared to controls; this is equivalent to 21% of the observed effect (1.67%/8%). The rest of the effect (100%-19%-21%=60% of the additional cardiomyocytes observed after therapy with CDCs) should be attributed to host tissue preservation (Supp Fig 11). Using the approach described above, we calculated the relative contributions of cardiomyocyte proliferation versus cardiomyogenic differentiation of host stem cells in myocyte replenishemnt post-MI to be 29% and 71% respectively; after CDC therapy, the relative contributions to regenerative cardiomyogenesis were 47% for cardiomyocyte proliferation and 53% for differentiation of progenitors (Fig 10A). If all cases of binucleation/ polyploidization are considered as instances where cell-cycle is activated abortively, then 51.7% of the observed DNA synthesis in controls and 70.9% in CDC-treated animals should be attributed to new myocyte generation. In this case, cardiomyogenic differentiation of recruited stem cells would result in generation of 6.1%x70.9%x39%x1.08% (since CDC treated hearts had

8% more myocytes) – 3.1%x51.7%x29%=1.36% more myocytes in CDC-treated hearts compared to controls; this is equivalent to 17% of the observed effect (1.36%/8%). Host tissue preservation would account for 72% (100%-11%-17%) of the effect. The relative contributions of cardiomyocyte proliferation versus cardiomyogenic differentiation of host stem cells in myocyte replenishemnt post-MI were 22% and 78% respectively; after CDC therapy, the relative contributions to regenerative cardiomyogenesis were 39% for cardiomyocyte proliferation and 61% for differentiation of progenitors.

Third, immunocytochemistry of isolated cells showed that 1.51% of resident GFP+ cardiomyocytes were BrdU+ at 5 weeks compared to 0.51% in controls. If all observed DNA synthesis translated to resident cardiomyocyte proliferation, CDC-induced cardiomyocyte cycling would result in generation of 1.51x1.08% (since CDC treated hearts had 8% more myocytes) – 0.51% = 1.12% more myocytes in CDC treated hearts compared to controls; this is equivalent to 14% of the observed effect (1.12%/8%). If all cases of binucleation/ polyploidization represent instances where cell-cycle is activated abortively in resident cardiomyocytes, then only 34.7% of the observed DNA synthesis in controls (see above) and 50% in CDC-treated animals (see above) should be attributed to new myocyte generation. In this case, CDC-induced cardiomyocyte cycling would result in generation of 1.51x50%x1.08% – 0.51%x34.7% = 0.64% more myocytes in CDC-treated hearts compared to controls; this is equivalent to 8% of the observed effect (0.64%/8%). We followed the same approach to quantify the contribution of stem cells to the myocyte pool. Immunocytochemistry of GFP- cardiomyocytes demonstrated that 7.2% (an additional 5.7% compared to the GFP+ fraction) of GFP-/áSA+ cardiomyocytes were BrdU+ 5 weeks after CDC treatment, compared to 3.1% (an additional 2.6%

compared to the GFP+ fraction) in infarcted controls. In addition, GFP- myocytes accounted for 29% and 39% of total border zone myocytes after MI and MI+CDCs respectively. Based on the above, if all observed DNA synthesis translated new myocyte formation, CDC-induced host stem cell myocyte replenishment re-entry would account for generation of 5.7%x39%x1.08% (since CDC treated hearts had 8% more myocytes) – 2.6%x29% = 1.65% more myocytes in CDC treated hearts compared to controls; this is equivalent to 20% of the observed effect (1.65%/8%). The rest of the effect (100%-14%-20%=66% of the additional cardiomyocytes observed after therapy with CDCs) should be attributed to host tissue preservation. If all cases of binucleation/ polyploidization are considered as instances where cell-cycle is activated abortively, then 51.7% of the observed DNA synthesis in controls and 70.9% in CDC-treated animals should be attributed to new myocyte generation. In this case, cardiomyogenic differentiation of recruited stem cells would result in generation of 5.7%x70.9%x39%x1.08% (since CDC treated hearts had 8% more myocytes) – 2.6%x51.7%x29%=1.3% more myocytes in CDC-treated hearts compared to controls; this is equivalent to 16% of the observed effect (1.3%/8%). Host tissue preservation would account for 76% (100%-8%-16%) of the effect.

Fourth, , histology in the infarct border zone showed that 5.5% of resident GFP+ cardiomyocytes were BrdU+ at 5 weeks compared to 1.6% in controls. If all observed DNA synthesis translated to resident cardiomyocyte proliferation (and taking into account that: a) border zone comprised 25% of total heart mass in our study; b) no differences in cardiomyocyte cycling were observed in the remote area; c) GFP+ myocytes accounted for 71% and 61% of total border zone myocytes after MI and

MI+CDCs respectively, ie MI hearts had 16% more GFP+ cardiomyocytes in the infarct border zone) CDC-induced cardiomyocyte cycling would result in generation of 5.5%x0.25x1.08% (since CDC treated hearts had 8% more myocytes) – 1.6%x0.25x1.16 = 1.02% more myocytes in CDC treated hearts compared to controls; this is equivalent to 13% of the observed effect (1.09%/8%). If all cases of binucleation/ polyploidization represent instances where cell-cycle is activated abortively in resident cardiomyocytes, then only 34.7% of the observed DNA synthesis in controls (see above) and 50% in CDC-treated animals (see above) should be attributed to new myocyte generation. In this case, CDC-induced cardiomyocyte cycling would result in generation of 5.5x0.25x50%x1.08% – 1.6%x0.25x34.7%x1.16 = 0.57% more myocytes in CDC-treated hearts compared to controls; this is equivalent to 7% of the observed effect (0.57%/8%). We followed the same approach to quantify the contribution of stem cells to the myocyte pool. Histology in the border zone demonstrated that 12.9% (an additional 7.4% compared to the GFP+ fraction) of GFP-/áSA+ cardiomyocytes were BrdU+ 5 weeks after CDC treatment, compared to 6.7% (an additional 5.1% compared to the GFP+ fraction) in infarcted controls. In addition, GFP- myocytes accounted for 29% and 39% of total border zone myocytes after MI and MI+CDCs respectively. Based on the above, if all observed DNA synthesis translated new myocyte formation, CDC-induced host stem cell myocyte replenishment re-entry would account for generation of 7.4%x39%x1.08% (since CDC treated hearts had 8% more myocytes) – 5.1%x29% = 1.63% more myocytes in CDC treated hearts compared to controls; this is equivalent to 20% of the observed effect (1.63%/8%). The rest of the effect (100%-13%-20%=67% of the additional cardiomyocytes observed after therapy with CDCs) should be attributed to

host tissue preservation. If all cases of binucleation/ polyploidization are considered as instances where cell-cycle is activated abortively, then 51.7% of the observed DNA synthesis in controls and 70.9% in CDC-treated animals should be attributed to new myocyte generation. In this case, cardiomyogenic differentiation of recruited stem cells would result in generation of 7.4%x70.9%x39%x1.08% (since CDC treated hearts had 8% more myocytes) – 5.1%x51.7%x29%=1.43% more myocytes in CDC-treated hearts compared to controls; this is equivalent to 18% of the observed effect (1.43%/8%). Host tissue preservation would account for 75% (100%-7%-18%) of the effect.

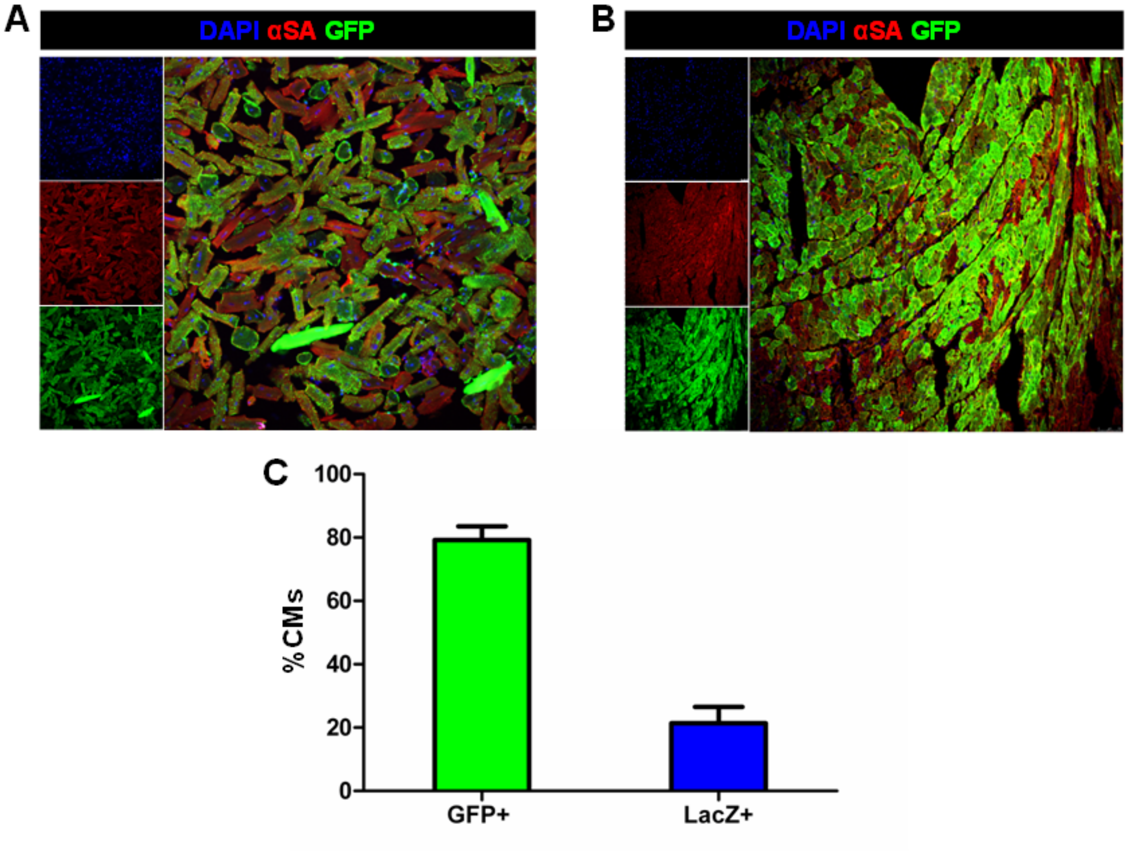

Supp Fig 1. A,B: 4-OH Tamoxifen pulsing of MerCreMer/ZEG mice results in efficient and specific labeling of cardiomyocytes by GFP, as determined by immunocytochemistry of dissociated myocytes (**A**) and confirmed by immunohistochemistry (**B**) (scale bars: 20μm) . **C**: ~80% of resident cardiomyocytes express GFP. All error bars represent SDs.

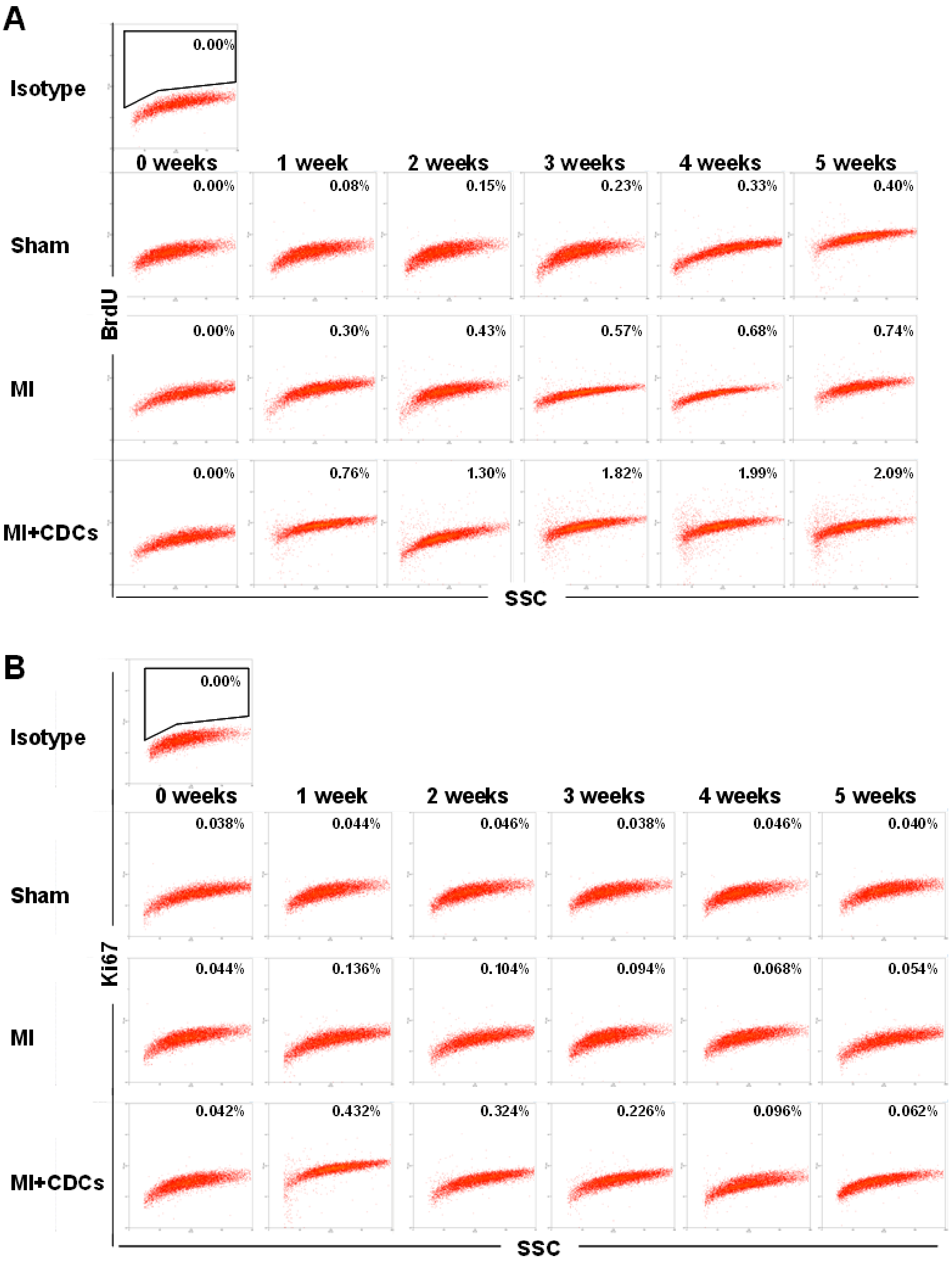

Supp Fig 2. A: Flow cytometric analysis of FACS-sorted GFP+ cardiomyocytes for BrdU incorporation. Representative images at all time points along with an isotype

control are shown. Numbers represent means of BrdU+ FACS-sorted cells at each timepoint. **B**: Flow cytometric analysis of FACS-sorted GFP+ cardiomyocytes for Ki67 expression. Representative images at all timepoints along with an isotype control are shown. Numbers represent means of Ki67+ FACS-sorted cells at each timepoint.

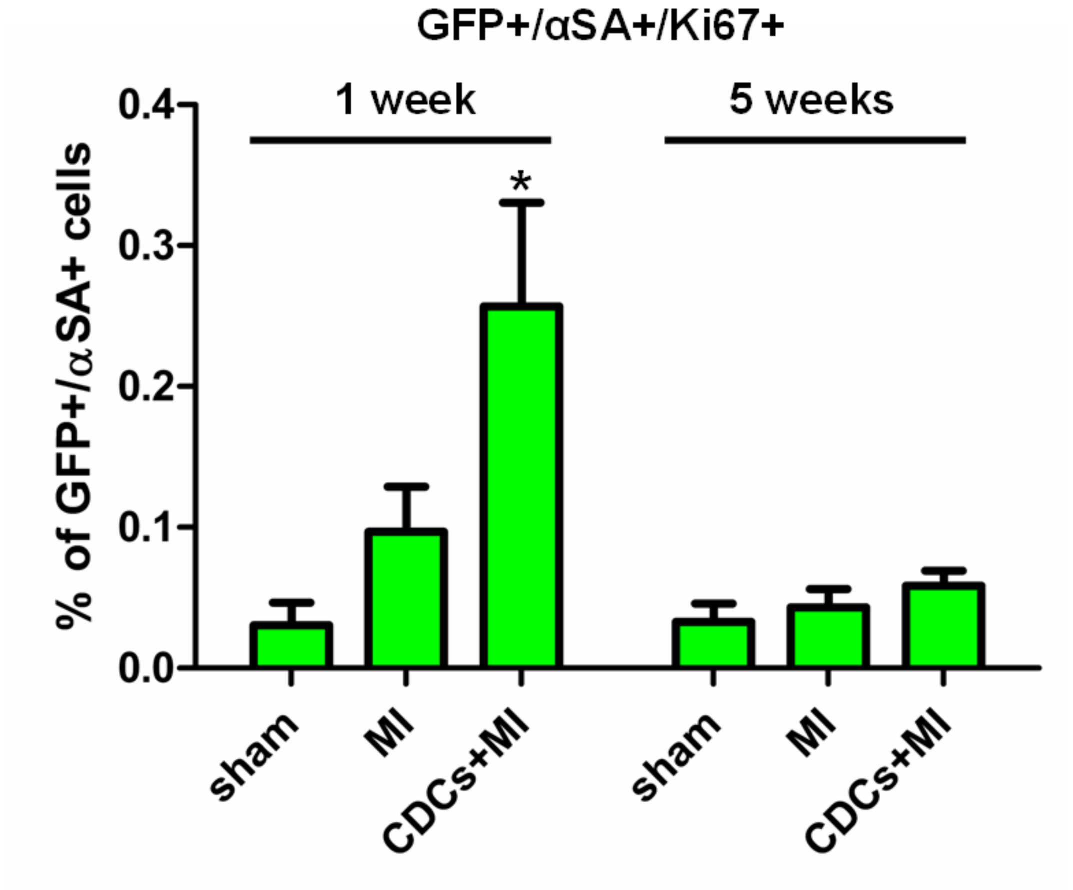

Supp Fig 3: Immunocytochemistry of enzymatically-dissociated cardiomyocytes isolated from hearts of bitransgenic mice that did not receive BrdU for GFP, αSA and Ki67. Comparison with Fig 3B (of the main manuscript) reveals similar percentages of Ki67+/GFP+ cardiomyocytes at 1 and 5 weeks in mice that received and did not receive BrdU, suggesting no significant effect of long-term BrdU administration on the cycling rates of resident cardiomyocytes (*$p<0.05$ compared to MI, sham; n=3/timepoint/group). All error bars represent SDs. One-way ANOVA followed by LSD post hoc test and independent samples t-test were used for statistical analysis (1w: CDCs vs sham p=0.001, CDCs vs MI p=0.006; all other p=ns).

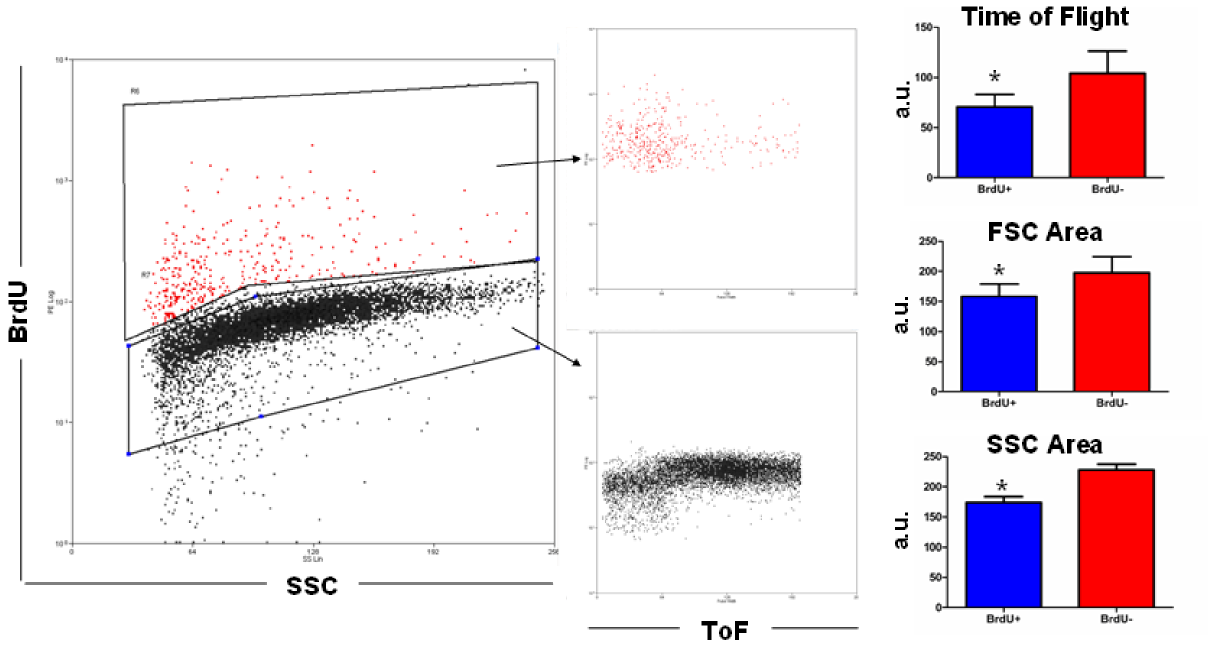

Supp Fig 4. Flow cytometric analysis of BrdU+/GFP+ cells (red dots) revealed that cycling endogenous cardiomyocytes were smaller (decreased Time of Flight [p=0.001], decreased Forward scatter area [p=0.002]) and less granular/complex (decreased side scatter area [p<0.001]) compared to non-cycling endogenous cardiomyocytes (BrdU-/GFP+; black dots). Color gating has been applied to the image (*p<0.05 compared to BrdU-/GFP+ cells). Independent samples t-test was used for statistical analysis. All error bars represent SDs.

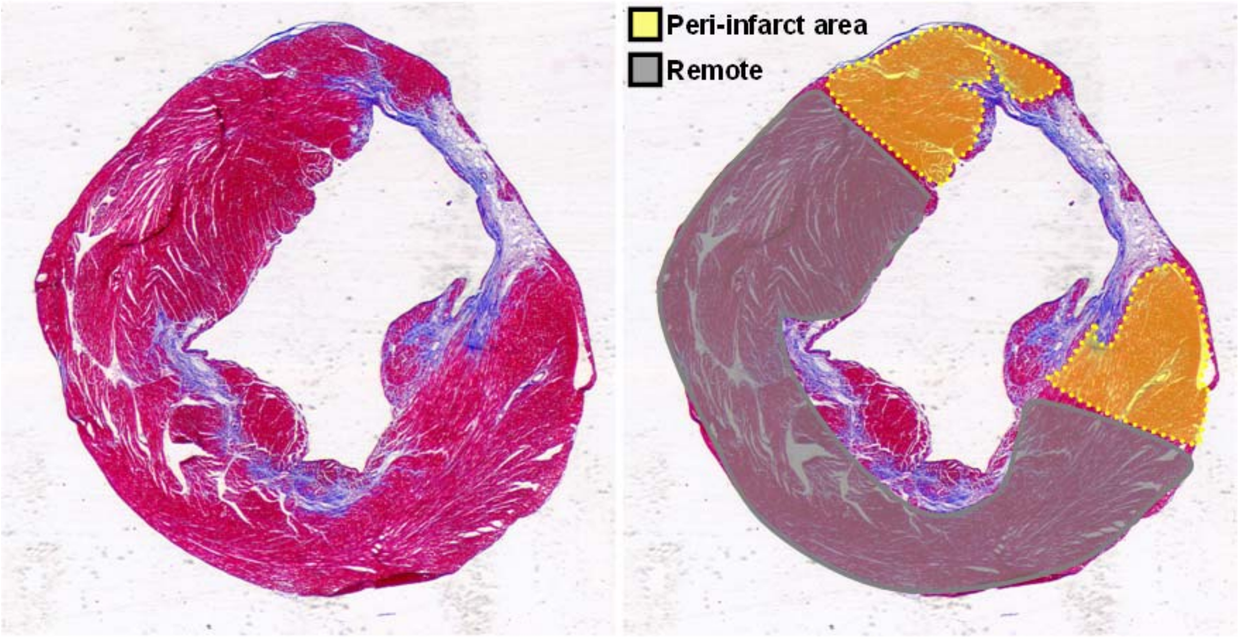

Supp Fig 5. Definition of peri-infarct area and remote myocardium. Peri-infarct area was defined as the area within one low-power field (10x) from the edges of the scar, but not including the scar. Peri-infarct area comprised ~25% of the non-infarcted viable myocardium in our study.

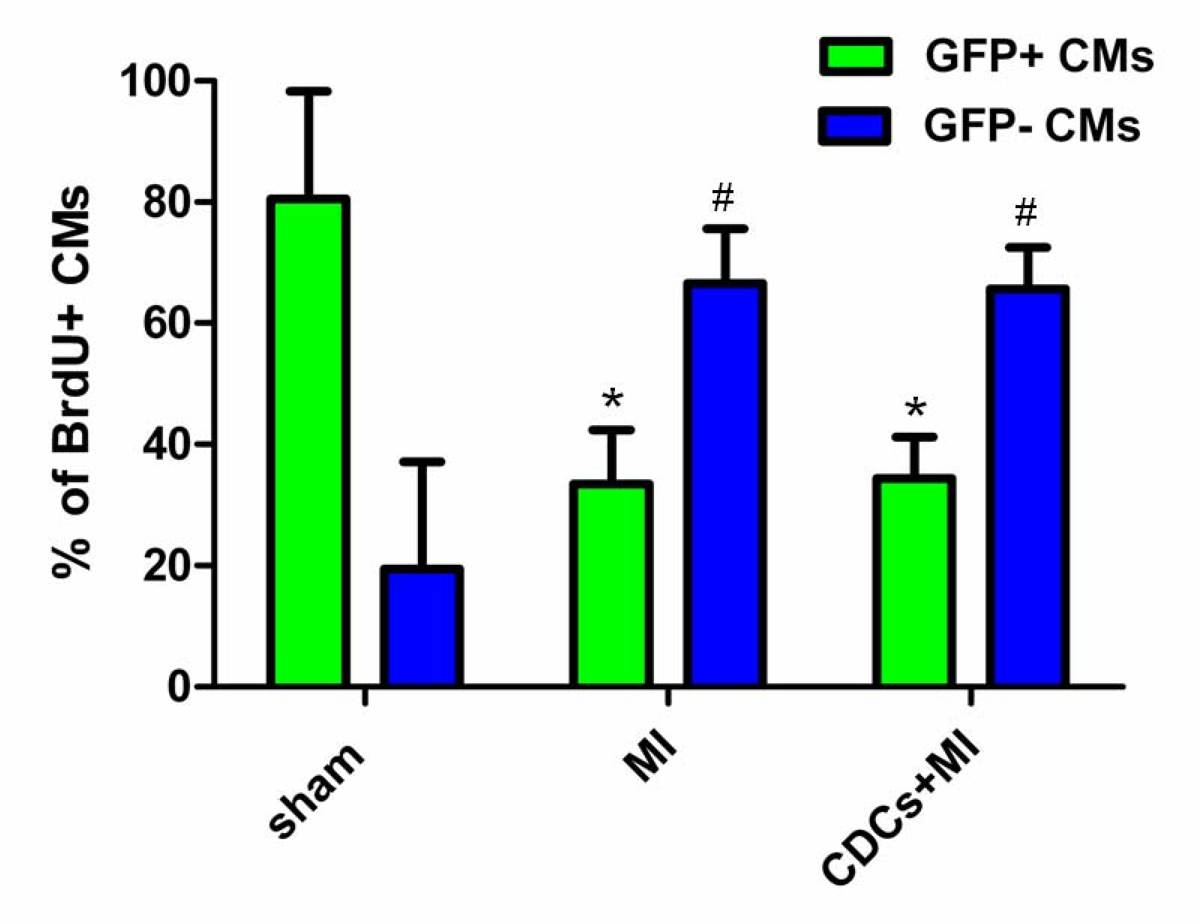

Supp Fig 6. Frequency of GFP expression in BrdU+ cardiomyocytes as determined by immunocytochemistry of dissociated cardiomyocytes for GFP, αSA and BrdU. The observed decrease in the frequency of GFP expression in BrdU+ cardiomyocytes after MI ($p<0.001$) and after MI+CDC ($p<0.001$) therapy is consistent with cardiomyogenic differentiation of recruited progenitors (*$p<0.05$ compared to sham GFP+ CMs, #$p<0.05$ compared to sham GFP- CMs; n=5/group). Independent samples t-test was used for statistical analysis. All error bars represent SDs.

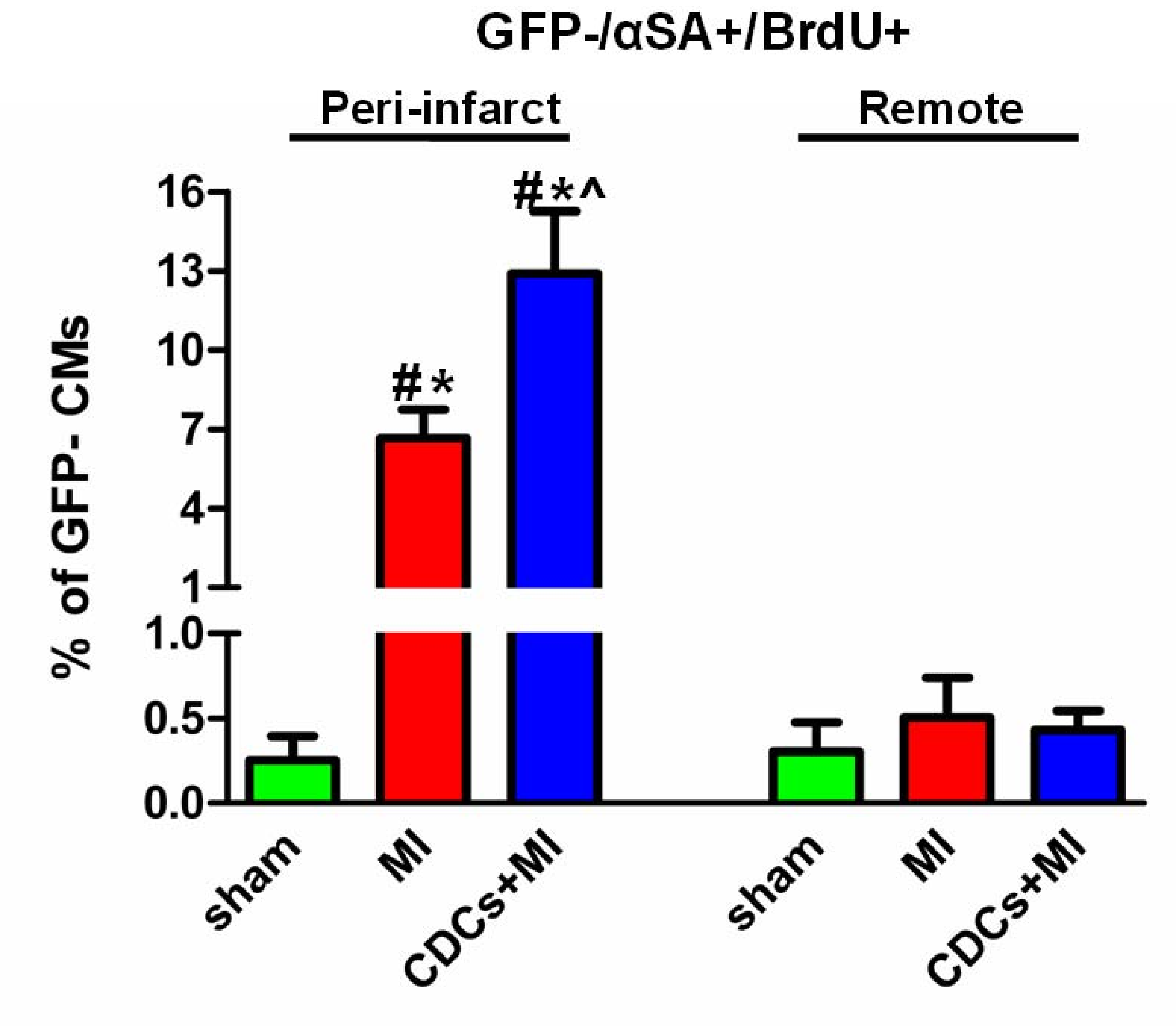

Supp Fig 7. Quantification of the rates of BrdU+/GFP- cardiomyocytes by immunohistochemistry in the peri-infarct area and the remote myocardium shows recruitment and cardiomyogenic differentiation of endogenous progenitors post-MI in border zone that is upregulated after CDC therapy (* $p<0.05$ compared to remote, # $p<0.05$ compared to sham, ^ $p<0.05$ compared to MI, n=3-5/group). One-way ANOVA followed by LSD post hoc test and independent samples t-test were used for statistical analysis (MI vs sham p=0.002CDCs vs sham $p<0.001$; CDCs vs MI p=0.002; MI peri-infarct vs remote p=0.008; CDCs peri-infarct vs remote p= 0.001; all other p=ns). All error bars represent SDs.

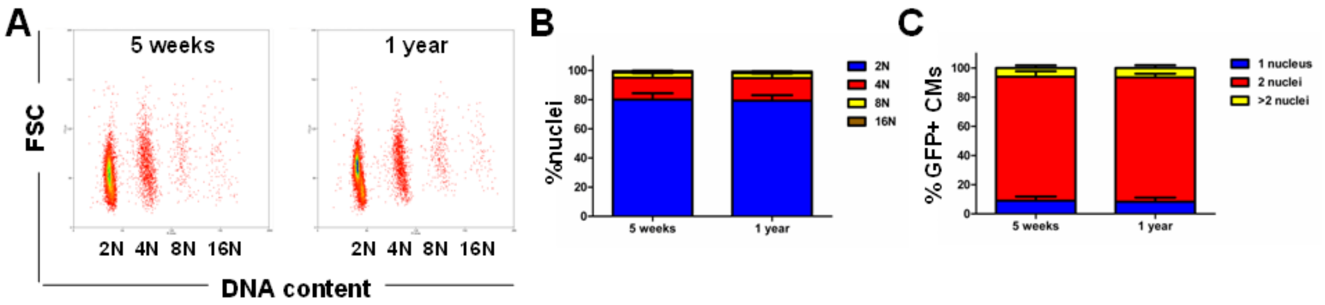

Supp Fig 8. **A,B**: Flow cytometric analysis of isolated nuclei from GFP+ FACS-sorted cells obtained from 5-week old and 1 year-old mice for measurement of DNA content. No differences in ploidy levels could be detected between young and old mice. **C**: Relative percentages of mononucleated, binucleated and multinucleated GFP+/αSA+ cells in young and old mice as determined by immunocytochemistry of enzymatically dissociated cardiomyocytes; no differences could be observed. Independent samples t-test was used for statistical analysis. All error bars represent SDs.

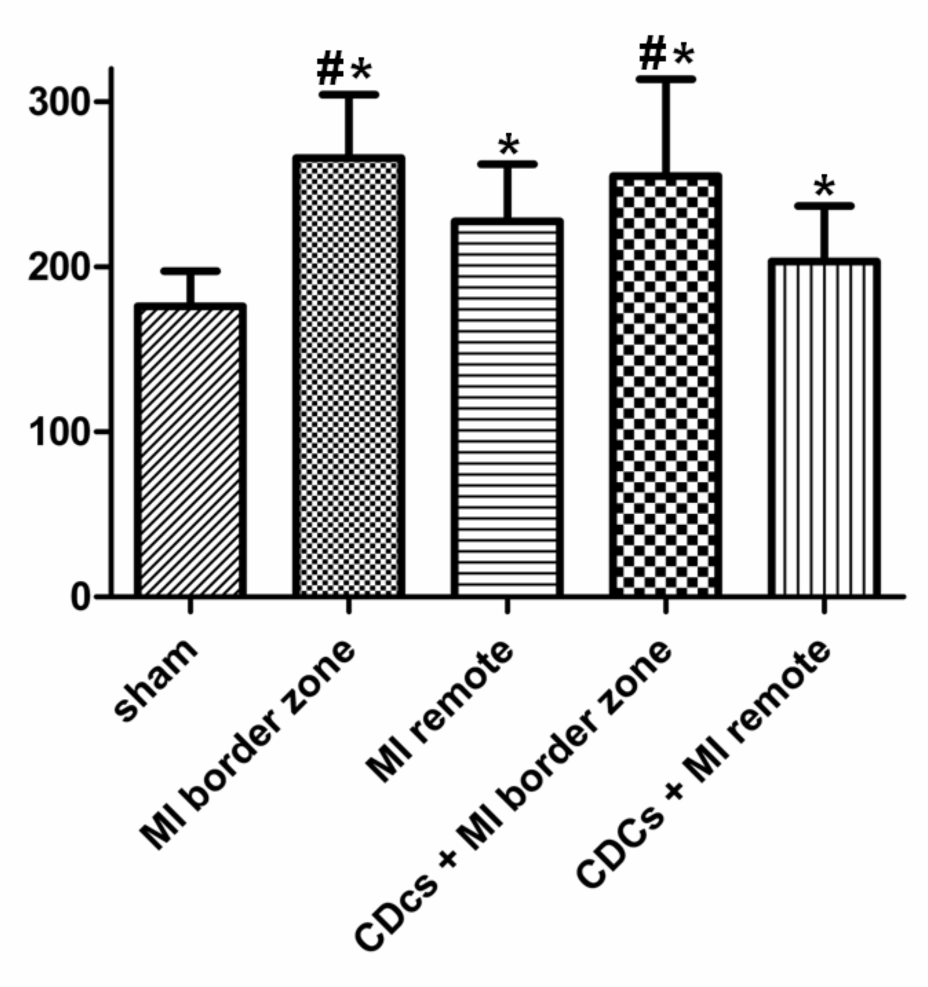

Supp Fig 9. Cross-sectional area of cardiomyocytes by immunohistochemistry. The cross-sectional area of cardiomyocytes located in the peri-infarct area (of both CDC-treated animals and infarcted controls) was higher compared to that of cardiomyocytes in remote myocardium. Cardiomyocytes from both the peri-infarct and remote myocardium had significantly higher cardiomyocyte area compared to cardiomyocytes in non-infarcted hearts (* $p<0.05$ compared to sham, # $p<0.05$ compared to remote, n=5-10/group). One-way ANOVA followed by LSD post hoc test was used for statistical analysis (MI border vs sham $p<0.001$; CDC border vs sham $p<0.001$; MI remote vs sham $p=0.005$; CDC remote vs sham $p=0.048$; MI border vs MI remote $p=0.034$; CDC border vs CDC remote $p=0.005$; all other p=ns). All error bars represent SDs.

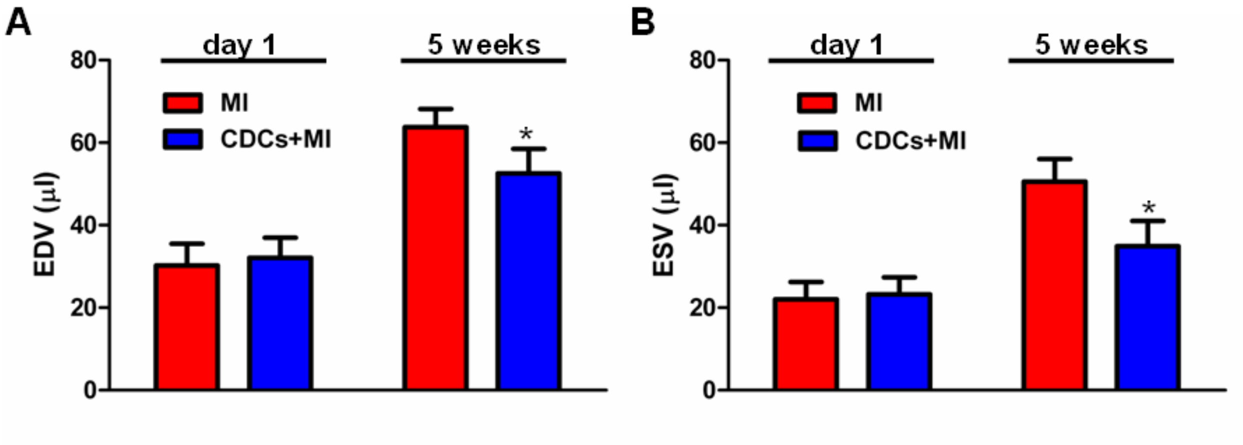

Supp Fig 10. Echocardiographic assessment of cardiac volumes. End-diastolic volume ($p<0.001$) (**A**) and end-systolic volume ($p<0.001$) (**B**) were smaller in CDC-treated hearts at 5 weeks, suggesting attenuation of adverse remodeling (* $p<0.05$ compared to MI, n=9/group). Independent samples t-test was used for statistical analysis. All error bars represent SDs.

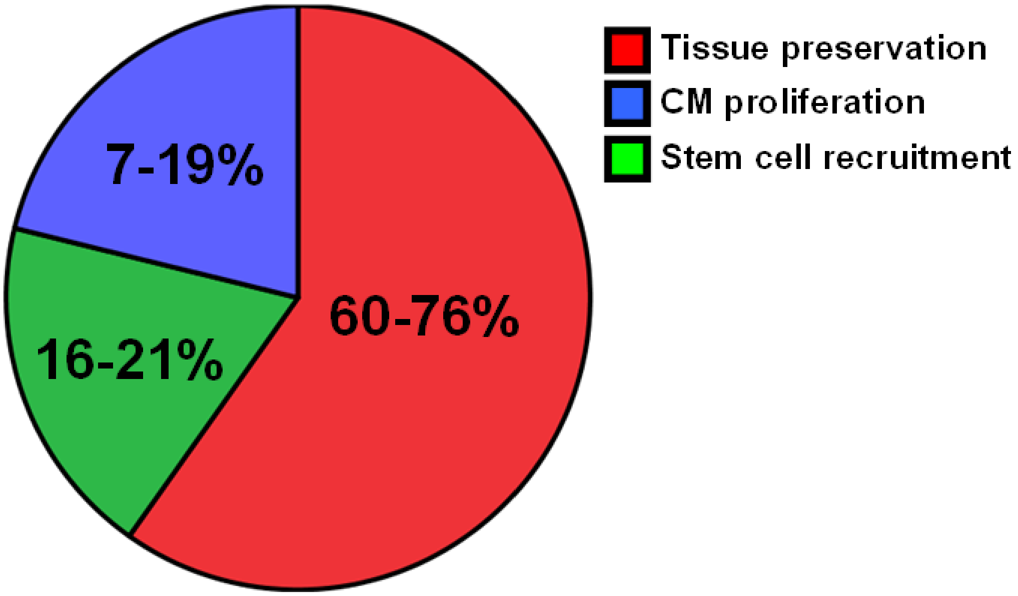

Supp Fig 11. Relative contributions of tissue preservation cardiomyocyte proliferation and cardiomyogenic differentiation of recruited endogenous stem cells to the increased viable myocardium observed after CDC therapy (n=5).

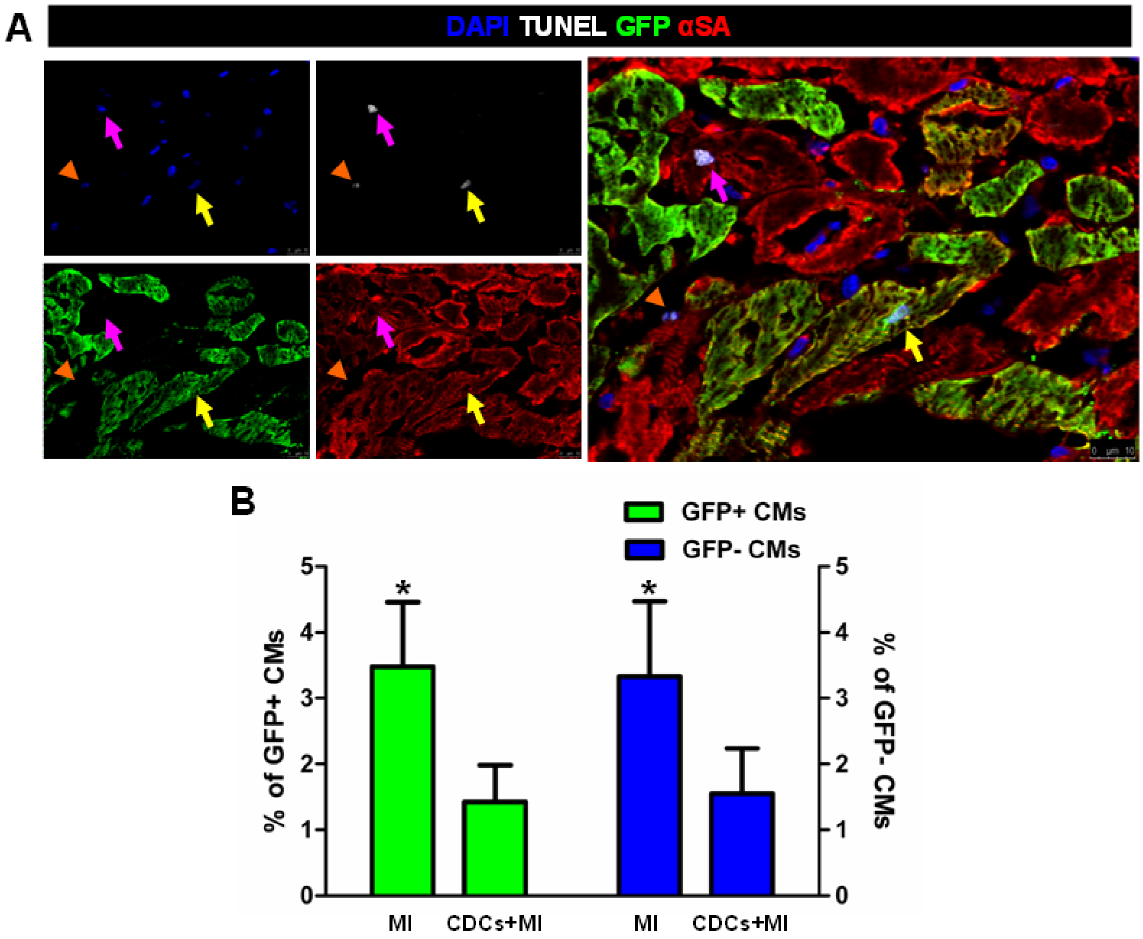

Supp Fig 12. Quantification of cardiomyocyte apoptosis by TUNEL staining 2 days post-MI. Yellow arrow shows an apoptotic GFP+ cardiomyocyte, purple arrow shows an apoptotic GFP- cardiomyocyte, orange arrowhead shows an apoptotic non-cardiomyocyte. Cardiomyocyte apoptosis was reduced in CDC-treated animals compared to infarcted controls. No difference in the rates of cardiomyocyte apoptosis could be detected between GFP+ and GFP- cardiomyocytes (* $p<0.05$ compared to CDC+MI, n=4/group). Independent samples t-test was used for statistical analysis (GFP+ CMs: MI vs CDCs $p=0.011$; GFP- CMs: MI vs CDCs $p=0.046$). All error bars represent SDs.
